# Minimum combined sleep, physical activity, and nutrition variations associated with lifeSPAN and healthSPAN improvements: a population cohort study

**DOI:** 10.1016/j.eclinm.2025.103741

**Published:** 2026-01-13

**Authors:** Nicholas A. Koemel, Raaj K. Biswas, Matthew N. Ahmadi, Armando Teixeira-Pinto, Mark Hamer, Leandro F.M. Rezende, John Mitchell, Rebecca M. Leech, Mouna Sawan, Margaret Allman-Farinelli, Dorothea Dumuid, Adrian Bauman, Carol Maher, Stephen Barrett, Clara Chow, Alice A. Gibson, David Raubenheimer, Samantha L. Hocking, Kathryn Williams, Peter A. Cistulli, Stephen J. Simpson, Emmanuel Stamatakis

**Affiliations:** a Mackenzie Wearables Research Hub, Charles Perkins Centre, University of Sydney, Sydney, NSW, Australia; b School of Health Sciences, Faculty of Medicine and Health, University of Sydney, Sydney, NSW, Australia; c Charles Perkins Centre, University of Sydney, Sydney, NSW, Australia; d Centre for Precision Data Science, University of Sydney, Sydney, NSW, Australia; e School of Public Health, Faculty of Medicine and Health, University of Sydney, Sydney, NSW, Australia; f Division of Surgery and Interventional Science, Institute Sport Exercise and Health, University College London, London, United Kingdom; g National Institute for Health Research, University College London, London, United Kingdom; h Department of Preventive Medicine, Chronic Disease Epidemiology Research Center, Escola Paulista de Medicina, Universidade Federal de São Paulo, São Paulo, Brazil; i Faculty of Health Sciences, Universidad Autónoma de Chile, Providencia, 7500912, Chile; j Institute for Physical Activity and Nutrition (IPAN), School of Exercise and Nutrition Sciences, Deakin, Geelong, VIC, Australia; k School of Pharmacy, Faculty of Medicine and Health, University of Sydney, Sydney, NSW, Australia; l School of Nursing, Faculty of Medicine and Health, University of Sydney, Sydney, NSW, Australia; m Alliance for Research in Exercise, Nutrition and Activity, Allied Health and Human Performance, University of South Australia, Adelaide, SA, Australia; n Bendigo Health and Holsworth Research Initiative, La Trobe Rural Health School, Bendigo, VIC, Australia; o Westmead Applied Research Centre, Faculty of Medicine and Health, University of Sydney, Sydney, NSW, Australia; p Leeder Centre for Health Policy, Economics and Data, Sydney School of Public Health, Faculty of Medicine and Health, University of Sydney, Sydney, NSW, Australia; q School of Life and Environmental Sciences, Faculty of Science, University of Sydney, Sydney, NSW, Australia; r Metabolism and Obesity Service, Royal Prince Alfred Hospital, Sydney, NSW, Australia; s Central Clinical School, Faculty of Medicine and Health, University of Sydney, Sydney, NSW, Australia; t Department of Endocrinology, Nepean Hospital, Kingswood, Sydney, NSW, Australia; u Department of Respiratory and Sleep Medicine, Royal North Shore Hospital, Sydney, NSW, Australia; v Northern Clinical School, Faculty of Medicine and Health, University of Sydney, Sydney, NSW, Australia

**Keywords:** Healthspan, Lifespan, Sleep, Nutrition, Diet, Physical activity, Exercise, Lifestyle risk factors, Life expectancy, Disease free life expectancy

## Abstract

**Background:**

Sleep, physical activity, and nutrition (SPAN) are key determinants of both life expectancy (lifespan) and disease-free life expectancy (healthspan), yet are often studied in isolation. This study aimed to determine the minimum combined SPAN improvements needed for a longer lifespan and healthspan.

**Methods:**

This prospective cohort comprised 59,078 participants from the UK Biobank, recruited between 2006 and 2010 (median age: 64.0 years; 45.4% male). Between 2013 and 2015, a subsample of participants was invited to wear a wrist worn accelerometer for 7 days. Moderate to vigorous physical activity (MVPA; mins/day) and sleep (hours/day) were calculated using a validated wearables-based algorithm. Diet was assessed using a 10-item diet quality score (DQS), including intake of vegetables, fruits, grains, meats, fish, dairy, oils, and sugar-sweetened beverages (ranging 0–100; higher indicates better quality). Lifespan and healthspan (free of cardiovascular disease (CVD), cancer, type II diabetes, chronic obstructive pulmonary disease (COPD), and dementia) were estimated across 27 joint tertile SPAN combinations and a composite SPAN score using life tables.

**Findings:**

Over an 8.1-year median follow-up, 2458 deaths, 9996 CVD, 7681 cancers, 2971 type II diabetes, 1540 COPD, and 508 dementia events occurred. Compared to the least favourable tertiles, the optimal tertiles (7.2–8.0 h/day of sleep; >42 min/day of MVPA; DQS of 57.5–72.5) had 9.35 additional years of lifespan (95% CI: 6.67, 11.63) and 9.45 years of healthspan (95% CI: 5.45, 13.61). Compared to the 5th percentile, a minimum combined improvement of 5 min/day of sleep, 1.9 min/day MVPA, and a 5-point increase in DQS (e.g., additional ½ serving of vegetables/day or additional 1.5 servings of whole grains per day) was associated with 1 additional year of lifespan (95% CI: 0.69, 1.15). For healthspan, a combined improvement of 24 min/day of sleep, 3.7 min/day of MVPA, and a 23-point DQS increase was associated with 4.0 additional years (95% CI: 0.50, 8.61).

**Interpretation:**

Modest concurrent improvements in sleep, physical activity, and diet were associated with meaningful gains in lifespan and healthspan.

**Funding:**

Australian National Health and Medical Research Council.

**Research in context**

**Evidence before this study**

Poor sleep, physical activity, and nutrition (SPAN) patterns are established modifiable causes of premature mortality and non-communicable disease. As major behaviour change is hard to both initiate and sustain, it is unsurprising that the current uni-disciplinary approach has had very limited success to date. Small changes across multiple SPAN behaviours may be more behaviourally sustainable, compared to large changes in each single SPAN behaviour. However, a uni-disciplinary research and policy mindset means that SPAN behaviours have traditionally been researched and promoted in isolation from each other without recognition of their behavioural interdependence or their synergistic effects on health outcomes.

**Added value of this study**

To our knowledge, our study is the first of its kind to investigate the minimum combined doses of device-measured sleep and physical activity, alongside a comprehensive dietary score for nutrition, associated with meaningful improvements in lifespan and healthspan. We show that while individual SPAN behaviours required substantial amounts to achieve improvements in lifespan and healthspan, when addressed in combinations, the overall dose needed for meaningful improvements was substantially lower. A minimum combined dose of an additional 5 min/day of sleep, 2 min/day of MVPA, and 5 DQS (e.g., ½ serving of vegetables per day or 1.5 servings of whole grains per day) was associated with one additional year of lifespan. In contrast, an additional 24.0 min/day of sleep, 4 min/day of MVPA and an improvement of 23 DQS points (e.g., additional 1 cup of vegetables, one serving of whole grains per day, and two servings per week of fish) were associated with 4 additional years of healthspan.

**Implications of all the available evidence**

This study demonstrates that small, concurrent improvements in sleep, physical activity, and diet quality were associated with clinically meaningful theoretical gains in lifespan and healthspan. These findings inform future trials and public health interventions by highlighting a pragmatic approach to improving population health that involves combined modest behavioural changes.

## Introduction

Sleep, physical activity, and nutrition (SPAN) are key modifiable risk factors for noncommunicable diseases (NCD) and all-cause mortality.^1^, ^2^, ^3^ Both insufficient and excessive sleep have been shown to disrupt normal metabolic and brain health through mechanisms such as impaired glycaemic control, inflammation, and dysregulation of appetite hormones.^4^ Physical inactivity contributes to one in six deaths in the UK^5^ and plays a major role in the progression of age-related chronic diseases. Poor diet quality and excess caloric intake adversely affect cardiometabolic health and are central to the global obesity epidemic.^6^^,^^7^

Collectively, SPAN behaviours are linked with increased risk of leading causes of NCD-related morbidity and mortality, including cardiovascular disease (CVD), certain cancers, type II diabetes, dementia, and respiratory conditions such as chronic obstructive pulmonary disease (COPD).^8^ Despite increases in life expectancy (lifespan) over recent decades,^9^ healthspan (defined as years lived free of major chronic disease) has remained stagnant or declined.^10^ In the UK, it is projected that by 2035, over two-thirds of adults aged ≥35 years will be living with multiple chronic conditions,^11^ likely resulting in greater healthcare expenditure and reduced quality of life.^12^

Modifying SPAN behaviours presents a viable strategy to extend healthspan by influencing the trajectory of biological ageing and delaying the onset of chronic diseases. However, these behaviours are often examined independently, neglecting the complex behavioural and physiological interdependencies between them.^4^^,^^13^^,^^14^ For instance, inadequate sleep duration may impair appetite regulation, leading to higher energy intake,^4^ while inadequate diet quality may reduce sleep quality. Similarly, sleep deprivation may increase fatigue and reduce physical activity.^4^ Emerging evidence supports a synergistic relationship among SPAN behaviours. A recent prospective study of over 59,000 UK adults^15^ found that modest concurrent improvements in all three behaviours were associated with meaningful reductions in mortality risk. A theoretical minimum combined increase of approximately 15 min/day of sleep, 1.6 min/day of moderate-to-vigorous physical activity (MVPA), and a 5-point increase in diet quality score (e.g., an additional ½ serving of vegetables per day or one fewer serving of processed meat per week) was associated with a 10% reduction in all-cause mortality risk. In isolation, achieving this level of risk reduction required 60% more sleep (additional 24 min/day), 25% more MVPA (2 min/day), while diet alone was unable to achieve this level of risk reduction.

Most prior studies examining lifestyle behaviours in relation to life expectancy^16^, ^17^, ^18^, ^19^, ^20^ and healthspan^21^, ^22^, ^23^ have relied on broad, self-reported behavioural categories (e.g., active vs. inactive), limiting granularity and interpretability. To our knowledge, no study has examined the combined associations of SPAN behaviours with lifespan and healthspan using high-resolution, interpretable metrics (e.g., mins/day of activity; servings of fruits per day). In this study, we applied a multidimensional behavioural framework^15^ to estimate years of lifespan and healthspan gained from combined theoretical improvements in SPAN behaviours. Our primary goal was to estimate the minimum theoretical behavioural improvements needed for meaningful gains in lifespan and healthspan.

## Methods

### Study design and participants

We used data from the UK Biobank accelerometry sub-study,^24^ part of a larger cohort comprising 502,629 adults aged 40–69 recruited from 2006 to 2010. Participants completed touchscreen questionnaires for sociodemographic, lifestyle characteristics, and health status at recruitment.

### Ethics

All participants provided informed consent, and the ethical approval was completed by the UK National Health Service and National Research Ethics Service for the UK (No. 11/NW/0382).

### Procedures

#### Outcome ascertainment

Data on all-cause mortality were retrieved via linkage from the National Health Service Central Register and the National Records of Scotland with censoring for England and Wales, and Scotland up to November 30th, 2022. Incidence of five major noncommunicable diseases (CVD, cancer, type II diabetes, COPD, and dementia) was ascertained using inpatient hospital records and general practitioner records. Additional cancer data linkage was obtained through national cancer registries. A complete list of ICD-10 codes, outcome definitions, and more detailed censorship dates are provided in Supplementary Methods 1.

#### Assessment of sleep, physical activity, and nutrition

Between 2013 and 2015, a subsample of 103,684 participants was invited to wear a wrist worn accelerometer (Axivity AX3, York, UK) on their dominant wrist for 7 days.^24^ Participants were included in the analysis if they had valid data for at least three days (>16 h/day), including at least one weekend day.^25^, ^26^, ^27^ We excluded participants with no sleep data, faulty devices, poor calibration and self-reported inability to walk. Missing covariate data were handled by complete case analysis (Supplementary Figure 1).

Sleep duration (hours/day) was estimated using a validated algorithm based on wrist tilt angle variation.^28^ Moderate-to-vigorous physical activity (MVPA; mins/day) was calculated using a validated two-stage machine learning algorithm.^25^^,^^27^^,^^29^^,^^30^ The two-stage random forest activity classifier first classifies each 10 s window (epoch) as sedentary (lying or sitting still), stationary plus (active sitting, standing still, active standing), walking, or running and then classifies each of these as either light, moderate or vigorous intensity.^25^^,^^27^^,^^29^^,^^30^ Walking activities (gardening, active commuting, etc.) were classified by normalised gravitational units (g) where <100 milli g were classified as light intensity (<3 metabolic equivalent tasks (METs)), ≥100 milli g and <400 milli g were considered moderate-intensity physical activity (≥3 to <6 METs), and ≥400 milli g were considered vigorous-intensity PA (≥6 METs).^29^ The physical activity classifier has a high overall 84.6% accuracy across all behaviours.^25^^,^^30^ Additional details regarding the development, validation and performance of this schema are provided in Supplementary Methods 2.

Diet quality was assessed using a 29-item food-frequency questionnaire (FFQ) completed at recruitment (2006–2010) covering consumption of commonly eaten foods over the past 12 months. A validated Diet Quality Score (DQS)^15^ was computed, ranging from 0 (poorest diet) to 100 (healthiest diet), based on intake of vegetables, fruits, fish, dairy, whole grains, vegetable oils, refined grains, processed meats, unprocessed red meats, and sugar-sweetened beverages (Supplementary Table 1).

### Statistical analyses

#### SPAN and life expectancy (lifespan)

**Fig. 1:**
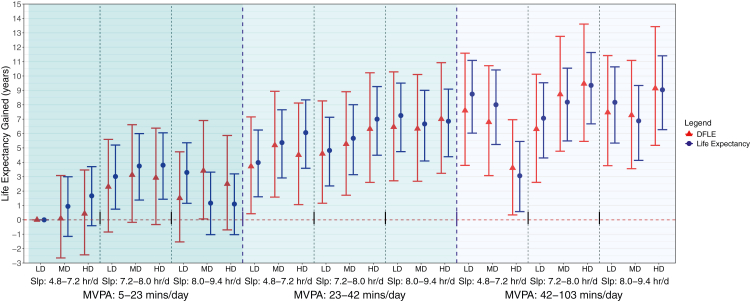
Multivariable-adjusted lifespan and healthspan associated with joint sleep, physical activity, and nutrition exposures (n = 59,078; all-cause mortality events = 2458). Legend: Life expectancy (lifespan) was estimated using life table models, with predictions derived from hazard ratio-adjusted mortality rates derived from the association between each mutually exclusive sleep, physical activity, and nutrition (SPAN) category and all-cause mortality. Disease-free life expectancy (DFLE; healthspan) was calculated as the expected lifespan free from cardiovascular disease, cancer, type II diabetes, chronic obstructive pulmonary disease, or dementia. DFLE incorporated a life table approach that included age-specific incidence rates for each condition. The all-cause mortality model is adjusted for age, sex, ethnicity, smoking, education, Townsend deprivation index, alcohol, discretionary screen time (time spent watching TV or using the computer outside of work), light intensity physical activity, medication (blood pressure, insulin, and cholesterol), previous diagnosis of major cardiovascular disease (defined as disease of the circulatory system, arteries, and lymph, excluding hypertension), previous diagnosis of cancer, and familial history of cardiovascular disease and cancer. The specific ranges for each exposure included sleep duration as 4.8–7.2 h/day (low), 7.2–8.0 h/day (medium), and 8.0–9.4 h/day (high); MVPA time: 5–23 min/day (low), 23–42 min/day (medium), and 42–103 min/day (high); diet quality using DQS: 32.5–50.0 (low), 50.0–57.5 (medium), and 57.5–72.5 (high). Sleep (hours/day), physical activity (moderate to vigorous intensity – MVPA-minutes/day), and nutrition (Dietary Quality Score, DQS) were included in the model as a joint term. Dashed blue lines separate tertiles MVPA and dashed black lines separate tertiles of sleep. Sleep (Slp); Low Diet Quality (LD); Medium Diet Quality (MD); High Diet Quality.

We estimated lifespan using a conventional period life table approach, modelling a hypothetical cohort exposed to age-specific mortality rates.^9^^,^^31^ We adjusted the mortality rates using all-cause mortality risk estimates derived from a multivariable Cox proportional hazard model (Supplementary Methods 3). To minimise the influence of sparse or outlier data, we winsorised values below the 2.5 percentile and above the 97.5 percentile for all SPAN exposures.^15^^,^^25^^,^^27^^,^^32^ This approach replaces all values below the 2.5th percentile with the 2.5th percentile value and all values above the 97.5th percentile with the 97.5th percentile value, retaining the full analytic sample. SPAN behaviours were jointly modelled as a 27-category variable representing all tertile combinations of sleep, MVPA and diet.^15^ We visualised the multivariable-adjusted associations of SPAN exposures with lifespan and healthspan by plotting each of the 27 categories as a forest plot with the lowest tertile of each exposure used as the reference point (Fig. 1; event numbers shown in Supplementary Table 2). Models were adjusted for age, sex, ethnicity, smoking, education, Townsend deprivation index, alcohol, discretionary screen time, light intensity physical activity, medication (blood pressure, insulin, and cholesterol), previous diagnosis of major CVD, previous diagnosis of cancer, and familial history of CVD and cancer.^15^ Further details on the covariates are provided in Supplementary Table 3. The proportional hazards assumptions including proportional hazards over time over exposure strata and linearity of continuous covariates were satisfied and verified using Schoenfeld residuals and Martingale Residuals, respectively.

#### SPAN and disease-free life expectancy (healthspan)

As per previous work,^9^^,^^31^ healthspan was calculated as an extension of the original life expectancy life table approach that included age-specific baseline prevalence and incidence rates for each condition. We calculated the lifespan free from CVD, cancer, type II, dementia, and COPD. Parametric sampling-based Monte Carlo simulation (10,000 iterations) was applied to account for uncertainty in disease prevalence estimates.^21^^,^^23^ To reduce reverse causation, we excluded participants who died within the first year of follow-up.^15^^,^^26^

We assessed the potential synergistic effects of SPAN behaviours using the relative excess risk due to interaction (RERI), attributable proportion due to interaction (AP), and the synergistic effects index (S) for all-cause mortality and each chronic condition. These three indices estimate the contribution of synergistic interactions between exposures where an RERI or AP of 0 and an S value of 1 denote no interaction effect. In some cases, the RERI can include negative values which reflect a sub-additive (antagonistic relationship between the three exposures). To account for the low prevalence of certain noncommunicable diseases and data sparsity of some of the behaviours, we used non-parametric bootstrapping to estimate the 95% confidence intervals.^33^^,^^34^

#### Dose–response analyses and the composite SPAN score

To explore the minimum behaviour changes^15^ needed for meaningful improvements in lifespan and healthspan, we constructed a continuous composite SPAN score (range 0–100), with each SPAN behaviour equally weighted. Higher scores reflect closer alignment to the theoretically optimal dose for all-cause mortality (Supplementary Methods 4). For example, sleep observed a U-shaped relationship with all-cause mortality, so the contribution of sleep was calculated as the highest (i.e., healthiest) at the nadir of the curve (7.5 h per day) and lower standardised scores with deviation from this centre–point. The overall contribution of each behaviour was then interpreted using the hazard ratio estimates from the joint tertile-based SPAN model with all-cause mortality. The model estimates were used to derive behaviour-specific weights, which were then applied to transform the SPAN score into the incremental variations needed for improvements in lifespan and healthspan. The 5th percentile of the composite SPAN score was used as the reference point in the dose–response analyses, and the median was used in sensitivity analyses. We also completed additional stratified analyses for men and women^9^ using sex-specific and age-specific life tables. We created a heatmap correlogram to illustrate lifespan gains from incremental changes in each SPAN behaviour (Supplementary Figure 2).^15^ We adjusted for self-reported discretionary rather than device measured sedentary behaviour to avoid a high degree of multicollinearity across the time-based movement behaviours.^35^^,^^36^ No evidence of problematic multicollinearity was found between behaviours (Supplementary Table 4).

### Sensitivity analyses

We conducted several sensitivity analyses.

• Adjusted for additional sleep characteristics, including self-reported insomnia, snoring, chronotype (morning/evening person), and daytime sleepiness.
• To ensure findings were robust to the choice of dietary indicator, we repeated the main analyses using the proportion of dietary ultra-processed food as a marker of diet quality (Supplementary Table 5).
• Adjusted for total energy intake and excluded participants who reported sex-specific implausible energy intake.
• Excluded participants with poor self-rated health, smokers, participants with underweight BMI (<18.5), and those in the top 20th percentile of the frailty index.
• Excluded participants with deaths in the first three years of follow-up or pre-existing chronic diseases.
• We repeated the main analyses adjusting for BMI.
• Repeated the main analyses with no winsorisation for potential outlier or sparse data and employing alternative thresholds for winsorisation derived using Tukey Fences.^37^
• Repeated the main analyses including participants with missing covariate information (n = 853) using multiple imputation to generate the complete case datasets.^38^
• Repeating the main analysis with adjustment for season of accelerometry data collection.^39^^,^^40^

All analyses were conducted in R (version 4.4.2) using the survival (version 3.8.3), rms (version 8.0-0), and ggplot2 (version 3.5.2) packages. Reporting followed the Strengthening the Reporting of Observational Studies in Epidemiology (STROBE) guidelines.

### Role of the funding source

This study is funded by an Australian National Health and Medical Research Council (NHMRC) Investigator Grant (APP1194510). The funder had no specific role in any of the following study aspects: the design and conduct of the study; collection, management, analysis, and interpretation of the data; preparation, review, or approval of the manuscript; and the decision to submit the manuscript for publication.

## Results

### Sample

The core analytical sample comprised 59,078 participants (median age [IQR]: 64.0 [7.8] years; 45.4% male). Over a median follow-up of 8.1 years, 2458 deaths, 9996 incident CVD, 7681 incident cancer, 2971 incident type II diabetes, 1540 incident COPD, and 508 incident dementia events occurred. Additional participant characteristics are provided in Supplementary Table 6.

### Combined SPAN associations with lifespan and healthspan

A near-linear trend was observed across joint SPAN tertile categories, where higher levels of sleep, MVPA, and diet quality score were associated with greater gains in lifespan and healthspan (Fig. 1). MVPA appeared to be the primary contributor to observed gains in life expectancy and healthspan.

Compared to the theoretically worst combination of behaviours (lowest combined tertile), the minimum SPAN combination associated with improvements of lifespan and healthspan became clear once entering the moderate MVPA category (i.e., >22 min/day), regardless of the corresponding level of sleep and DQS. Low MVPA, moderate sleep and high diet were associated with an additional 3.80 years of lifespan (95% CI: 1.43, 6.05) and 2.93 years of healthspan (95% CI: −0.32, 6.37). In contrast, moderate MVPA, moderate sleep, and high DQS were associated with an additional 7.00 years of lifespan (95% CI: 4.49, 9.26) and 6.32 years of healthspan (95% CI: 2.60, 10.22). The highest life expectancy gained was observed with high MVPA, moderate sleep, and high DQS, which was associated with an additional 9.35 years of lifespan (95% CI: 6.67, 11.63) and 9.46 years of healthspan (95% CI: 5.45, 13.61).

### Synergistic relationship of SPAN behaviours with healthspan and lifespan

There was evidence of a modest positive synergistic interaction between SPAN behaviours for all-cause mortality, supporting a greater than additive beneficial association with the three combined behaviours (RERI = 0.06; 95% CI: 0.005, 0.13; AP = 11.7; 95% CI: 1, 40; S = 0.89; 95% CI: 0.84, 0.97). We observed no evidence of a synergistic relationship between SPAN behaviours with healthspan (RERI = 0.009; 95% CI: −0.001, 0.02; AP = 1.0; 95% CI: −0.1, 3.6; S = 0.95; 95% CI: 0.93, 1.02). Estimates revealed a non-significant interaction for most individual conditions, although there was a borderline statistical significance for cancer suggesting a beneficial synergistic relationship between SPAN and cancer risk (RERI: 0.02; 95% CI: 0, 0.04; AP = 2.0; 95% CI: 0, 7.0; S = 0.92; 95% CI: 0.89, 1.00) (Supplementary Figures 3-4).

### Dose–response associations of individual and combined SPAN behaviours with lifespan

**Fig. 2:**
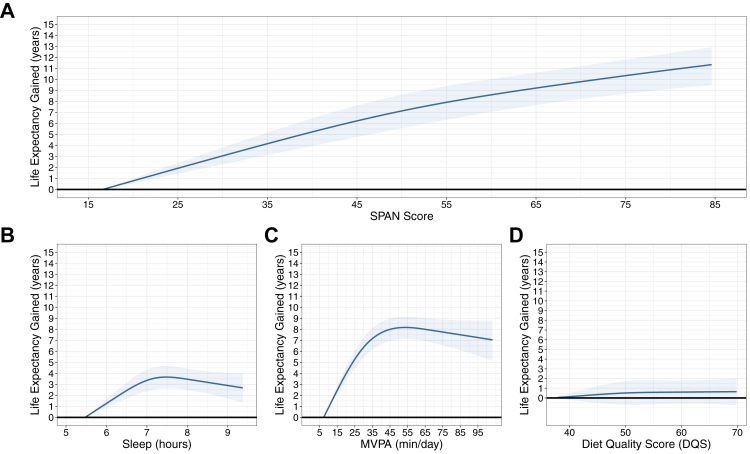
Multivariable-adjusted dose–response association between A) composite SPAN score B) sleep C) physical activity and D) nutrition with lifespan (n = 59,078; all-cause mortality events = 2458). Legend: Life expectancy (lifespan) was estimated using stratified sex-specific life table models, with predictions based on hazard ratio-adjusted mortality rates derived from the association between the composite sleep, physical activity, and nutrition (SPAN) score and all-cause mortality. The all-cause mortality model is adjusted for age, ethnicity, smoking, education, Townsend deprivation index, alcohol, discretionary screen time (time spent watching TV or using the computer outside of work), light intensity physical activity, medication (blood pressure, insulin, and cholesterol), previous diagnosis of major cardiovascular disease (defined as disease of the circulatory system, arteries, and lymph, excluding hypertension), previous diagnosis of cancer, and familial history of cardiovascular disease and cancer. The SPAN score is comprised of sleep (hours/day), physical activity (moderate to vigorous intensity – MVPA, minutes/day), and nutrition (Dietary Quality Score, DQS) were combined as continuous variables, each weighted equally, with scores ranging from 0 to 100. Higher scores indicated a more beneficial combined SPAN value and the referent point used was the 5th percentile of the composite score. The weighting of each exposure within the SPAN score was determined based on the theoretically optimal levels identified from the dose-response relationship with all-cause mortality. In figures B–D, the individual dose-response relationship between sleep, physical activity, nutrition and life expectancy was examined using the 5th percentile for each exposure as the referent point.

The dose–response association was found between the composite SPAN score and lifespan (Fig. 2A). Compared to the 5th percentile of the composite SPAN score, the median composite SPAN score (51.5) was associated with an additional 7.40 years of lifespan (95% CI: 5.81, 8.87), while a maximum score (84.7) yielded 11.34 years (95% CI: 9.49, 12.91). The estimates for the composite SPAN score and the individual behaviours stratified by sex and using the median value as the reference group are shown in Supplementary Figures 4-5.

Sleep showed an inverted U-shaped association with lifespan, plateauing around 7.5 h per day (3.39 years; 95% CI, 2.56–4.18: Fig. 2B). MVPA showed an inverted J-shaped association with lifespan, peaking at approximately 50 min/day (8.14 years; 95% CI, 7.11, 9.12; Fig. 2C). The association between diet quality score and lifespan was modest and not statistically significant (Fig. 2D).

### Minimal variations across the three SPAN behaviours and lifespan

Table 1 presents the effects of concurrent and individual theoretical improvements in SPAN associated with lifespan years gained. The first rows indicate the minimal differences in the combined SPAN behaviours associated with one year of lifespan gained. For example, compared to the combined SPAN reference point (5th percentile of all SPAN behaviours), an additional 5 min/day of sleep, 1.9 min/day MVPA, and increasing DQS by 5 points were associated with an additional 1 year of lifespan (95% CI: 0.50, 8.61). An increase of 60 min/day of sleep, 8.8 min/day MVPA, and 20 DQS points corresponds to a 5 year increase in lifespan (95% CI: 3.98, 5.98).

**Table 1:** Minimum concurrent variations in sleep, physical activity, and nutrition associated with increments of lifespan gained compared to 5th percentile of SPAN exposures.

Years of Lifespan Gained^a^	Additional Composite SPAN Score Dose	Additional Sleep (min/day)	Additional Physical Activity (min/day)	Additional Nutrition (DQS points)	Additional Sleep^b^ (min/day)	Additional Physical Activity^b^ (min/day)	Additional Nutrition^b^ (DQS points)
		Combined SPAN exposure			Individual SPAN exposures		
≈1 Year (0.92; 95% CI: 0.69, 1.15)	5	5	1.9	5	25	2.3	35.5^b^
≈2 Years (2.06; 95% CI: 1.54, 2.58)	9	15	3.8	5	49	4.5	–
≈3 Years (2.96; 95% CI: 2.22, 3.70)	13	30	5.4	10	77	6.7	–
≈4 Years (4.07; 95% CI: 3.06, 5.06)	18	45	7.3	10	–	9.1	–
≈5 Years (4.93; 95% CI: 3.72, 6.11)	22	60	8.8	20	–	11.7	–
≈6 Years (5.96; 95% CI: 4.54, 7.31)	27	75	11.0	20	–	14.8	–
≈7 Years (7.06; 95% CI: 5.50, 8.53)	33	90	13.0	30	–	19.4	–
≈8 Years (8.00; 95% CI: 6.42, 9.47)	39	120	15.4	30	–	47.1	–
≈9 Years (9.04; 95% CI: 7.51, 10.45)	47	150	18.4	35	–	–	–
≈10 Years (9.96; 95% CI: 8.38, 11.39)	55	180	24.9	35	–	–	–

Table 1 displays the minimum concurrent combinations of sleep, physical, activity and nutrition associated with increments of life expectancy gained (years) compared to individual sleep, physical activity, and nutrition (SPAN) exposures. The combined columns show the SPAN combinations and corresponding years of life expectancy gained compared to the 5th percentile of sleep (5.5 h/day), physical activity (7.3 min/day), and nutrition (36.9 DQS) in one year increments. For comparison, the dose needed for individual SPAN exposures is shown on the right side of the table. Empty cells denote that the individual SPAN exposure could not achieve that gain in life expectancy in isolation. All models were adjusted for age, sex, ethnicity, smoking, education, Townsend deprivation index, alcohol, discretionary screen time (time spent watching TV or using the computer outside of work), light intensity physical activity, medication (blood pressure, insulin, and cholesterol), previous diagnosis of major cardiovascular disease (defined as disease of the circulatory system, arteries, and lymph, excluding hypertension), previous diagnosis of cancer, and familial history of cardiovascular disease and cancer. Moderate to vigorous physical activity (MVPA); Diet quality score (DQS).

a Years of lifespan are reported from the point where clear, meaningful improvements were first observed, defined as where the association became significant.

b Symbol denotes individual behaviours were non-significant in the relationship with lifespan. Each exposure was adjusted by the median value of the other two SPAN exposures.

### Dose–response associations of individual and combined SPAN behaviours with healthspan

**Fig. 3:**
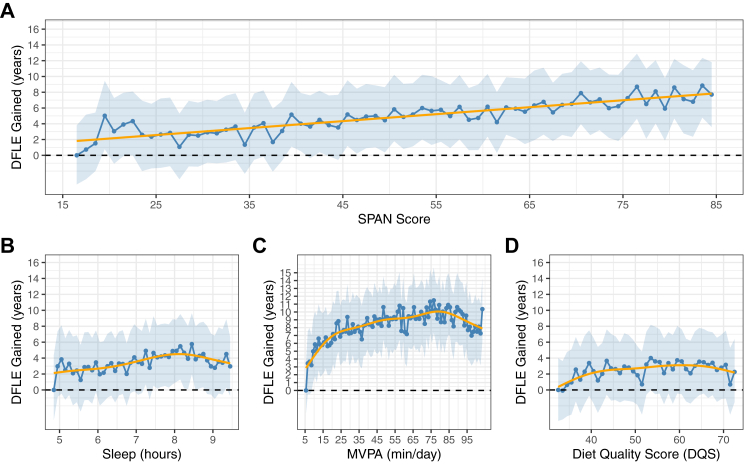
Multivariable-adjusted dose–response association between A) composite SPAN score B) sleep C) physical activity and D) nutrition with healthspan (n = 59,078; all-cause mortality events = 2458). Legend: Disease free life expectancy (DFLE; healthspan) was estimated using stratified life table models, with predictions based on hazard ratio-adjusted mortality rates derived from the association between the composite sleep, physical activity, and nutrition (SPAN) score and all-cause mortality. DFLE was calculated as an extension of the life table approach, accounting for the age-specific prevalence and incidence of the five leading contributors to disease burden, including cardiovascular disease, type II diabetes, cancer, and chronic obstructive pulmonary disease. The all-cause mortality model is adjusted for age, ethnicity, smoking, education, Townsend deprivation index, alcohol, discretionary screen time (time spent watching TV or using the computer outside of work), light intensity physical activity, medication (blood pressure, insulin, and cholesterol), previous diagnosis of major cardiovascular disease (defined as disease of the circulatory system, arteries, and lymph, excluding hypertension), previous diagnosis of cancer, and familial history of cardiovascular disease and cancer. The SPAN score is comprised of sleep (hours/day), physical activity (moderate to vigorous intensity – MVPA, minutes/day), and nutrition (Dietary Quality Score, DQS) were combined as continuous variables, each weighted equally, with scores ranging from 0 to 100. Higher scores indicated a more beneficial combined SPAN value, and the referent point used was the 5th percentile of the composite score. The weighting of each exposure within the SPAN score was determined based on the theoretically optimal levels identified from the dose-response relationship with all-cause mortality. In figures B–D, the individual dose-response relationship between sleep, physical activity, nutrition and life expectancy was examined using the 5th percentile for each exposure as the referent point.

The composite SPAN score was linearly associated with healthspan (Fig. 3A). The median SPAN score (51.5) was associated with an additional 4 years (4.05; 95% CI: 0.50, 8.61) of healthspan. Sleep duration was associated with subtle meaningful improvement in healthspan at 8 h/day, whereby compared to the 5th percentile (5.4 h per day) there was a gain in healthspan of 4 years (95% CI, 2.25, 4.87: Fig. 3B), although the improvements were nullified with durations beyond 8.5 h. Compared to the 5th percentile of MVPA, there was a J-shaped association between MVPA and healthspan, with gains plateauing at approximately 75 min/day associated with an additional 10 years of healthspan (95% CI, 5.86, 14.23 years gained; Fig. 3C). There was a subtle but non-statistically significant association between DQS and healthspan (Fig. 3D).

### Minimal combined variations across the three SPAN behaviours and healthspan

Table 2 presents the minimum theoretical combinations needed for meaningful healthspan gained. The relationship became statistically significant beyond approximately 45 points, which corresponds to nearly 4 years (4.05 years; 95% CI: 0.50, 8.61) of additional healthspan gained and achieving up to approximately 8 years (7.98 years; 95% CI: 3.47, 11.63) of additional healthspan at the maximum composite SPAN score. This minimum dose (42 points) of additional composite SPAN score is equivalent to a theoretical change of 24 min/day extra sleep, 3.7 min/day MVPA, and an improvement of 23 DQS points (e.g., an additional one cup of vegetables per day, one serving of whole grains per day, and two servings of fish per week). Among the individual SPAN behaviours, MVPA had the strongest relationship with healthspan (Fig. 3B), while sleep and DQS were associated with a maximum of approximately 4–5 years additional healthspan, but with uncertainty estimates that were not significant (Fig. 3B–D).

**Table 2:** Minimum concurrent variations in sleep, physical activity, and nutrition associated with increments of healthspan gained compared to 5th percentile of SPAN exposures.

Years of Healthspan Gained	Additional Composite SPAN Score Dose	Additional Sleep (min/day)	Additional Physical Activity (min/day)	Additional Nutrition (DQS points)	Additional Sleep (min/day)	Additional Physical Activity (min/day)	Additional Nutrition (DQS points)
		Combined SPAN exposure			Individual SPAN exposures		
≈1 Year (1.00; 95% CI: −4.07, 4.34)	0.5	0.5	0.1	0.5	2^b^	0.3^b^	3^b^
≈2 Years (1.96; 95% CI: −2.39, 5.37)	3	3	0.4	3	4^b^	0.5^b^	5^b^
≈3 Years (3.01; 95% CI: −1.15, 6.89)	14	13	2.1	13	108^b^	1.0^b^	34^b^
≈4 Years (4.05; 95% CI: 0.50, 8.61)	25^a^	24	3.7	23	189	3.9	–
≈5 Years (5.02; 95% CI: 1.40, 9.61)	37	35	5.5	33	–	6.8	–
≈6 Years (5.98; 95% CI: 1.66, 9.77)	48	46	7.1	43	–	10.2	–
≈7 Years (7.03; 95% CI: 4.54, 12.41)	60	57	8.9	54	–	15.1	–
≈8 Years (7.73; 95% CI: 3.48, 11.27)	68	65	10.0	61	–	29.6	–

Table 2 displays the minimum concurrent combinations of sleep, physical activity and nutrition associated with increments of disease-free life expectancy (healthspan) gained (years) compared to individual sleep, physical activity, and nutrition (SPAN) exposures. Disease-free life expectancy was calculated as an extension of the life table approach, accounting for the age-specific prevalence and incidence of the five leading contributors to disease burden, including cardiovascular disease, type II diabetes, cancer, and chronic obstructive pulmonary disease. The combined columns show the SPAN combinations and corresponding years of disease-free life expectancy gained compared to the 5th percentile of sleep (5.5 h/day), physical activity (7.3 min/day), and nutrition (36.9 DQS) in one-year increments. For comparison, the dose needed for individual SPAN exposures is shown on the right side of the table. Empty cells denote that the individual SPAN exposure could not achieve that gain in healthspan in isolation. All models were adjusted for age, sex, ethnicity, smoking, education, Townsend deprivation index, alcohol, discretionary screen time (time spent watching TV or using the computer outside of work), light intensity physical activity, medication (blood pressure, insulin, and cholesterol), previous diagnosis of major CVD (defined as disease of the circulatory system, arteries, and lymph, excluding hypertension), previous diagnosis of cancer, and familial history of CVD and cancer. Moderate to vigorous physical activity (MVPA); Diet quality score (DQS).

a The point where clear, meaningful improvements were first observed for healthspan, defined as where the association became significant.

b Symbol denotes individual behaviours were non-significant in the relationship with healthspan. Each exposure was adjusted by the median value of the other two SPAN exposures.

### Sensitivity analyses

Excluding individuals with poor baseline health (n = 51,164; events = 1887), early mortality (n = 58,610; events = 1990), or pre-existing disease (n = 51,166; events = 1888); adjusting for BMI (n = 58,363; events = 2405); controlling for sleep characteristics (n = 37,475; events = 1506) or total energy intake (n = 42,990; 1758 events); and using an alternative diet quality metric (n = 41,936; events = 1758) did not materially alter results (Supplementary Figures 6–13). The overall interpretation of the results remained consistent when using alternative winsorisation thresholds, excluding participants with potential sparse or outlier data, and repeating the main analysis with multiple imputation for missing covariate data (n = 59,931; events = 2505), and adjustment for season of accelerometry data collection (Supplementary Figures 14–17).

## Discussion

In this large prospective cohort study using a multi-behaviour analytical framework,^15^ we provide the first investigation into the minimum combined doses of device-measured sleep and physical activity, alongside a comprehensive dietary score for nutrition, associated with meaningful improvements in lifespan and healthspan. We showed that while individual SPAN behaviours required substantial amounts to achieve improvements in lifespan and healthspan, when addressed in combinations, the overall dose needed for meaningful improvements was substantially lower. Compared to the 5th percentile of all SPAN behaviours, a minimum combined dose of an additional 5 min/day of sleep, 1.9 min/day of MVPA, and 5 DQS (e.g., 1/2 serving of vegetables per day or additional 1.5 servings of whole grains per day) was associated with one additional year of lifespan. Larger theoretical changes in SPAN behaviours were associated with statistically significant improvements in healthspan, i.e., an additional 24 min/day of sleep, 3.7 min/day of MVPA and an improvement of 23.0 DQS points (e.g., an additional one cup of vegetables per day, one serving of whole grains per day, and two servings of fish per week) were associated with 4 additional years of healthspan.

Our findings build upon previous studies from the United States,^17^ UK^22^ and China^31^ showing that adherence to multiple low-risk lifestyle behaviours (smoking cessation, maintaining a healthy weight, improved diet quality, and reduced alcohol intake) can increase lifespan by 8–14 years and extend healthspan by 9–10 years for women and men, respectively.^22^ However, prior studies^16^^,^^18^, ^19^, ^20^, ^21^^,^^23^ relied almost exclusively on self-reported questionnaire data, which were often assessed in isolation, and have not explored synergistic interactions between behaviours. Here, using device-based measurements for sleep and physical activity in combination with a comprehensive diet quality index, we showed the most optimal SPAN behavioural combinations linked to nearly a decade of additional lifespan and healthspan.

Our findings for physical activity are consistent with previous UK Biobank data, which have shown a gain of approximately 2 years of additional years of lifespan in those self-reporting high levels of total physical activity (≥3000 MET-min per week,^41^ equivalent to approximately 12.5 h of brisk walking or 5 h of running). In contrast, meeting the physical activity guidelines (≈22 min/day of MVPA) through device-based measures was associated with a 5-year gain in life expectancy, consistent with our findings (≈19 min/day of MVPA in isolation).^41^ Our results reinforce growing evidence of the foundational role of sleep in metabolic health,^4^ showing a maximum gain of ≈4 years additional lifespan and healthspan when sleeping ≈8 h/day compared to the 5th percentile (5 h/day). While dietary associations remained subtle and non-significant in isolation, the combination of diet with sleep and physical activity suggests it plays an important synergistic role, as supported by the RERI.

A central finding of our study is the demonstration of a modest synergistic association of SPAN behaviours with lifespan. This is biologically plausible and grounded in the unique interconnected physiological pathways between these behaviours,^4^^,^^13^^,^^14^ including energy regulation and metabolic adaptations.^4^ When considered individually, substantially higher levels of each behaviour were required to achieve a similar extension in lifespan, or the improvements were not possible at all. For example, to theoretically gain one additional year of lifespan would require 25 min of additional sleep per day, with a maximum lifespan gain of three years. In combination, however, relatively modest theoretical changes, such as 5 additional mins/day of sleep, 1.9 min/day of MVPA, and half a serving of vegetables per day, were associated with a meaningful extension of lifespan by one year. For healthspan, although there was no formal statistical evidence of a synergistic SPAN relationship, physical activity required exponentially higher doses to achieve the same healthspan benefit as the combined SPAN behaviours. For example, 30 min/day MVPA was necessary to achieve 8 additional years free of chronic disease vs. 10 mins/day MVPA in combination with sleep (65 additional mins/day) and dietary improvements (61 additional DQS points). These findings suggest that leveraging the synergy of combined SPAN behaviours may enable new opportunities for feasible and sustainable approaches for improving lifespan and healthspan in adults who may lack the time, motivation, or financial resources to make substantial lifestyle changes.

To our knowledge, this is the first study to apply a high-resolution, device-based, multidimensional behavioural framework^15^ to estimate the minimum theoretical combined improvements in SPAN behaviours associated with meaningful gains of healthspan and lifespan years. Previous work exploring the role of lifestyle behaviours with lifespan^16^, ^17^, ^18^, ^19^, ^20^, ^21^, ^22^ has done so through broad self-reported categorical terms, limiting the translation into feasible and practical messaging for behaviour change. Modelling behavioural changes in interpretable units (e.g., mins/day, servings/week) provides actionable insights for public health messaging and behavioural interventions. By including participants from the UK Biobank's wearables sub-study, we were able to incorporate device-based estimates for both sleep and physical activity, offering a more accurate and granular assessment of these behaviours. However, we acknowledge that these estimates represent theoretical extrapolations from our multivariable restricted cubic spline model rather than direct observations. Additional studies are needed to examine the translation of these findings into practice and determine the induction time needed to produce clinically meaningful improvements in healthspan and lifespan. The measurement source of sleep and physical activity also presents a limitation when making comparisons with nutrition, which was self-reported in this study. This methodological difference may partially explain why the findings differ from existing literature on the association between diet with lifespan and healthspan,^17^^,^^21^^,^^23^ as we observed a subtle non-significant relationship. This discrepancy may reflect underlying differences in measurement quality, where diet may have been more significantly impacted by regression dilution bias,^42^ attenuating its true association with mortality and morbidity. In the UK Biobank,^24^ there is also a temporal lag between the data collection of the behaviours, as FFQ dietary data were collected a median of 5.5 years before the wearable device measures of sleep and physical activity. The potential regression dilution bias and the lag-time between measures may have also contributed to the modest synergistic relationship observed for lifespan and unclear synergy for healthspan. Additionally, while this study examined the burden of diseases through prevalence and incidence of five major chronic conditions from available general practitioner or hospitalisation records, this approach may not fully reflect the true burden of these conditions. For example, chronic conditions such as type II diabetes, early-stage dementia, COPD, and some cancers may present clinical symptoms years prior to a formal diagnosis or hospitalisation event,^43^, ^44^, ^45^, ^46^ resulting in a potential overestimation of the years free from chronic conditions (i.e., healthspan) in the present study. The possibility of reverse causation or residual confounding cannot be eliminated, although we conducted a range of sensitivity analyses, including the exclusion of participants with a mortality event within the first three years of follow-up, self-rated poor health, high frailty index, underweight BMI, or a chronic condition at baseline. For example, prodromal symptoms of certain conditions, such as dementia,^47^ may emerge before diagnosis resulting in sleep disruption, reduced physical activity, and loss of appetite. We also note that the UK Biobank cohort is overall healthier than the general UK population, including lower prevalence of smoking, alcohol, obesity and less likely to live in socioeconomically disadvantaged areas which could influence the generalisability of these results to broader more diverse populations.^48^ However, our recent work has demonstrated that the lack of cohort representativeness may not materially distort the association between physical activity and diet with mortality.^49^ Further research is needed to examine how SPAN behaviours interact with other unhealthy behaviours, such as smoking and alcohol, in relation to lifespan and healthspan.

This study demonstrates that small, combined improvements in sleep, physical activity, and nutrition are associated with theoretical increases in both lifespan and healthspan that are clinically meaningful and relevant to population health. Much larger theoretical changes in each individual behaviour were associated with equivalent lifespan and healthspan gains, suggesting a potential synergy across behaviours. Our findings suggest that very small, likely achievable, combined changes in SPAN behaviours, may offer a powerful and feasible public health opportunity for improving lifespan for at least a year, while slightly larger behavioural changes may be required to stave off chronic disease altogether for several years.

## Contributors

NAK, RKB, MNA: conceptualization, methodology, software, formal analysis, validation, visualization, writing – original draft. NK and RKB had access to, and verified, the underlying data and analytical code. ATP, MH, LR, JM, RL, MS, MA, DD, AB, CM, SB, CC, AG, DR, SH, KW, PC, SS: conceptualization, methodology, writing – review and editing. ES: funding acquisition, project administration, resources, supervision, writing – review and writing. ES is the guarantor, is responsible for the overall content and accepts full responsibility for the work and/or the conduct of the study, had access to the data and controlled the decision to publish. All authors read and approved the final manuscript. The corresponding author attests that all listed authors meet authorship criteria and that no others meeting the criteria have been omitted.

## Data Sharing Statement

The data that support the findings of this study are available from the UK Biobank, but restrictions apply to the availability of these data, which were used under license for the current study and so are not publicly available. Data are, however, available from the authors upon reasonable request and with the permission of the UK Biobank.

## Declaration of interests

ES is a paid consultant and holds equity in Complement 1, a US-based company whose products and services relate to promotion of physical activity and other lifestyle behaviours.

RML has received an NHMRC Investigator Grant (2019–2024) and conference support from Deakin University and the NHMRC. RML serves as the Honorary Treasurer of the Nutrition Society of Australia. SLH has received honoraria from Lilly Australia, Novo Nordisk, Astra Zeneca, Amgen, Sanofi-Aventis, Nestlé Health Sciences, iNova, and Servier for lectures and manuscript writing.

SLH has received travel support from Lilly Australia, Novo Nordisk, Amgen, and CSL Seqirus, and serves on advisory boards for Lilly, Novo Nordisk, Ethicon, and Astra Zeneca. SLH also serves as President of the National Association of Clinical Obesity Services and as a Council Member of the Australian and New Zealand Obesity Society.

PC has received an NHMRC Investigator Grant and an academic chair from ResMed. PC receives royalties from UpToDate and Quintessence, and has served as a consultant for ResMed, SomnoMed, and Eli Lilly. PC has received lecture honoraria from SomnoMed and Eli Lilly, and equipment support from ResMed and SomnoMed.

KW has received a Novo Nordisk grant for biobanking, consulting fees from Lilly, Novo Nordisk, and Boehringer Ingelheim, and honoraria from Novo Nordisk and Boehringer Ingelheim for obesity-related presentations. KW has also received travel support from Novo Nordisk and Lilly for educational meetings within Australia, and medication donations from Novo Nordisk. KW serves as Clinical Lead of the Family Metabolic Health Service at Nepean Hospital, an Advisor for the Obesity Collective, and a Committee Member of the Australian and New Zealand Obesity Society.

MAF has received grants from the Australian Research Council, the National Health and Medical Research Council, Qantas Airways, and NSW Health. MAF received registration support from the Australian Academy of Science to attend the International Union of Nutrition Sciences meeting and receives honoraria for service on an NHMRC Committee.

DD has received an Australian Research Council Discovery Early Career Award and serves as President of the CoDa Association.

CC holds an NHMRC Investigator Grant and serves as an unpaid Board Member of both the National Heart Foundation and the Western Sydney Local Health District.

All other authors disclose no conflict of interest for this work.
