# Supplementary material for: Minimum combined sleep, physical activity, and nutrition variations associated with lifeSPAN and healthSPAN improvements: a population cohort study
Source: eClinicalMedicine. 2026 Jan 13;92:103741. doi: 10.1016/j.eclinm.2025.103741 (PMC12947648; doi:10.1016/j.eclinm.2025.103741)
Supplement: Supplementary Material [file mmc1.docx]

**ONLINE SUPPLEMENTARY MATERIAL**

**Minimum combined sleep, physical activity, and nutrition variations associated with lifeSPAN and healthSPAN improvements: a population cohort study**

| **Page** | **Item** |
| --- | --- |
| **3** | **Supplementary Figure 1:** Flow diagram of participants in the study |
| **4** | **Supplementary Figure 2:** Years of lifespan gained with concurrent variations in sleep, physical activity, and nutrition (n = 59,078; all-cause mortality events = 2,458) |
| **5** | **Supplementary Figure 3:** Synergistic relationship between sleep, physical activity, and nutrition in relation to mortality and morbidity |
| **6** | **Supplementary Figure 4:** Synergistic relationship between sleep, physical activity, and nutrition in relation to mortality and morbidity by individual diseases |
| **7** | **Supplementary Figure 5:** Multivariable-adjusted dose-response association between A) SPAN score B) sleep C) physical activity and D) nutrition with lifespan in males (n = 26,810; all-cause mortality events = 1,520) and females (n = 32,268; all-cause mortality events = 938) |
| **8** | **Supplementary Figure 6:** Multivariable-adjusted dose-response association between A) SPAN score B) sleep C) physical activity and D) nutrition with lifespan in males (n = 26,810; all-cause mortality events = 1,520) and females (n = 32,268; all-cause mortality events = 938) using alternative referent point |
| **9** | **Supplementary Figure 7:** Multivariable-adjusted associations of combined sleep, physical activity, and nutrition with lifespan and healthspan following exclusion of individuals with poor health (n = 51,164; events = 1,887) |
| **10** | **Supplementary Figure 8:** Multivariable-adjusted associations of combined sleep, physical activity, and nutrition with lifespan and healthspan excluding individuals with baseline chronic conditions (n = 51,166; events = 1,888) |
| **11** | **Supplementary Figure 9:** Multivariable-adjusted associations of combined sleep, physical activity, and nutrition with lifespan and healthspan excluding individuals with a mortality event in the first three years of follow-up (n = 58,610; events = 1,990) |
| **12** | **Supplementary Figure 10:** Multivariable-adjusted associations of combined sleep, physical activity, and nutrition with lifespan and healthspan adjustment for BMI (n = 58,363; events = 2,405) |
| **13** | **Supplementary Figure 11:** Multivariable-adjusted associations of combined sleep, physical activity, and nutrition with lifespan and healthspan adjustment for sleep characteristics (n = 37,475; events = 1,506) |
| **14** | **Supplementary Figure 12:** Multivariable-adjusted associations of combined sleep, physical activity, and nutrition with lifespan and healthspan using the proportion of ultra-processed food (n = 41,936; events = 1,758) |
| **15** | **Supplementary Figure 13:** Multivariable-adjusted associations of combined sleep, physical activity, and nutrition with lifespan and healthspan adjusted for total energy intake (n = 42,990; 1,758 events) |
| **16** | **Supplementary Figure 14:** Multivariable-adjusted associations of combined sleep, physical activity, and nutrition with lifespan and healthspan with no winsorisation (n = 59,078; all-cause mortality events = 2,458) |
| **17** | **Supplementary Figure 15:** Multivariable-adjusted associations of combined sleep, physical activity, and nutrition with lifespan and healthspan using Tukey’s Fences as an alternative winsorisation threshold (n = 59,078; all-cause mortality events = 2,458) |
| **18** | **Supplementary Figure 16:** Multivariable-adjusted associations of combined sleep, physical activity, and nutrition with lifespan and healthspan using an imputed data set for incomplete covariate data (n = 59,931; all-cause mortality events = 2,505) |
| **19** | **Supplementary Figure 17:** Multivariable-adjusted lifespan and healthspan associated with joint sleep, physical activity, and nutrition exposures adjusted for season of accelerometery data collection (n = 59,078; all-cause mortality events = 2,458) |
| **21** | **Supplementary Methods 1:** Additional study design details |
| **23** | **Supplementary Methods 2:** Wearable behaviour classification methods |
| **26** | **Supplementary Methods 3:** Calculation of lifespan and healthspan |
| **29** | **Supplementary Methods 4:** Calculation of the composite SPAN score |
| **30** | **Supplementary Table 1:** Diet quality score index for food-frequency questionnaire dietary data |
| **31** | **Supplementary Table 2:** Mortality and disease events across the mutually exclusive sleep, physical activity, and nutrition combinations |
| **32** | **Supplementary Table 3:** Covariate definitions |
| **34** | **Supplementary Table 4:** Model variance inflation factors for combined SPAN behaviours |
| **35** | **Supplementary Table 5:** NOVA classification of food groups for 24-hour dietary recall data |
| **36** | **Supplementary Table 6:** Participant Characteristics |

**
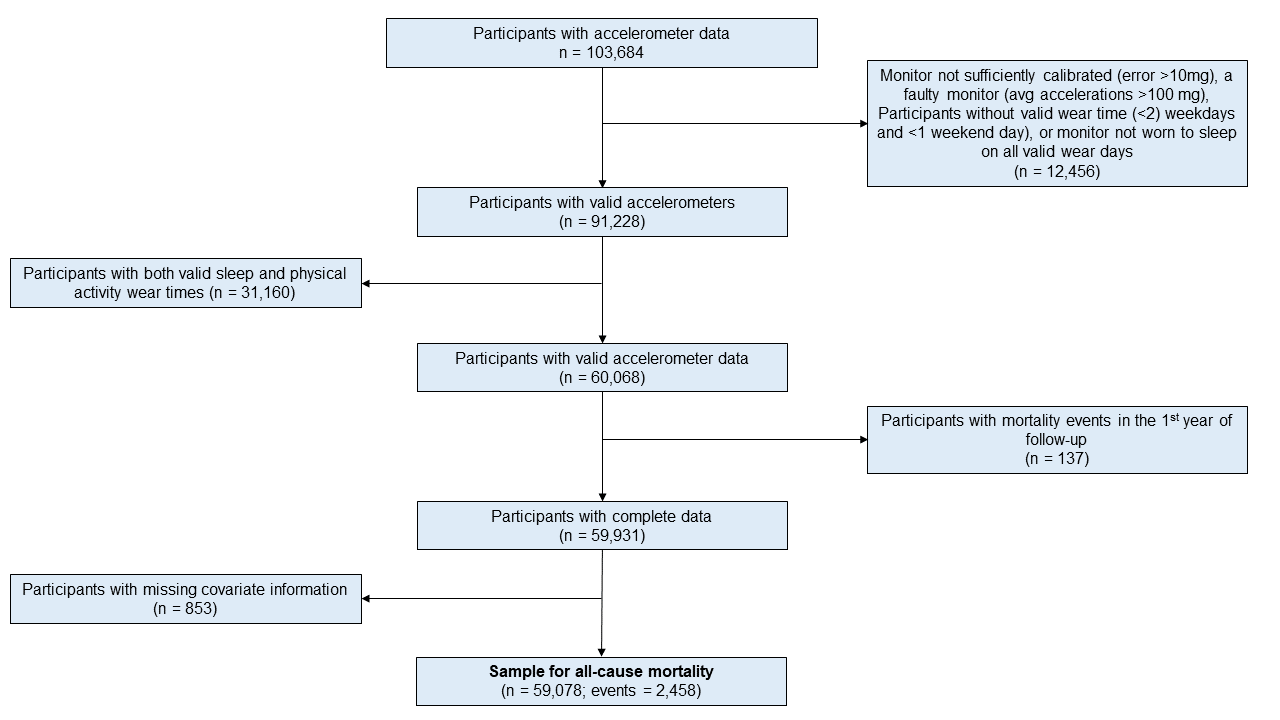
**

**Supplementary Figure 1.** Participant flow chart


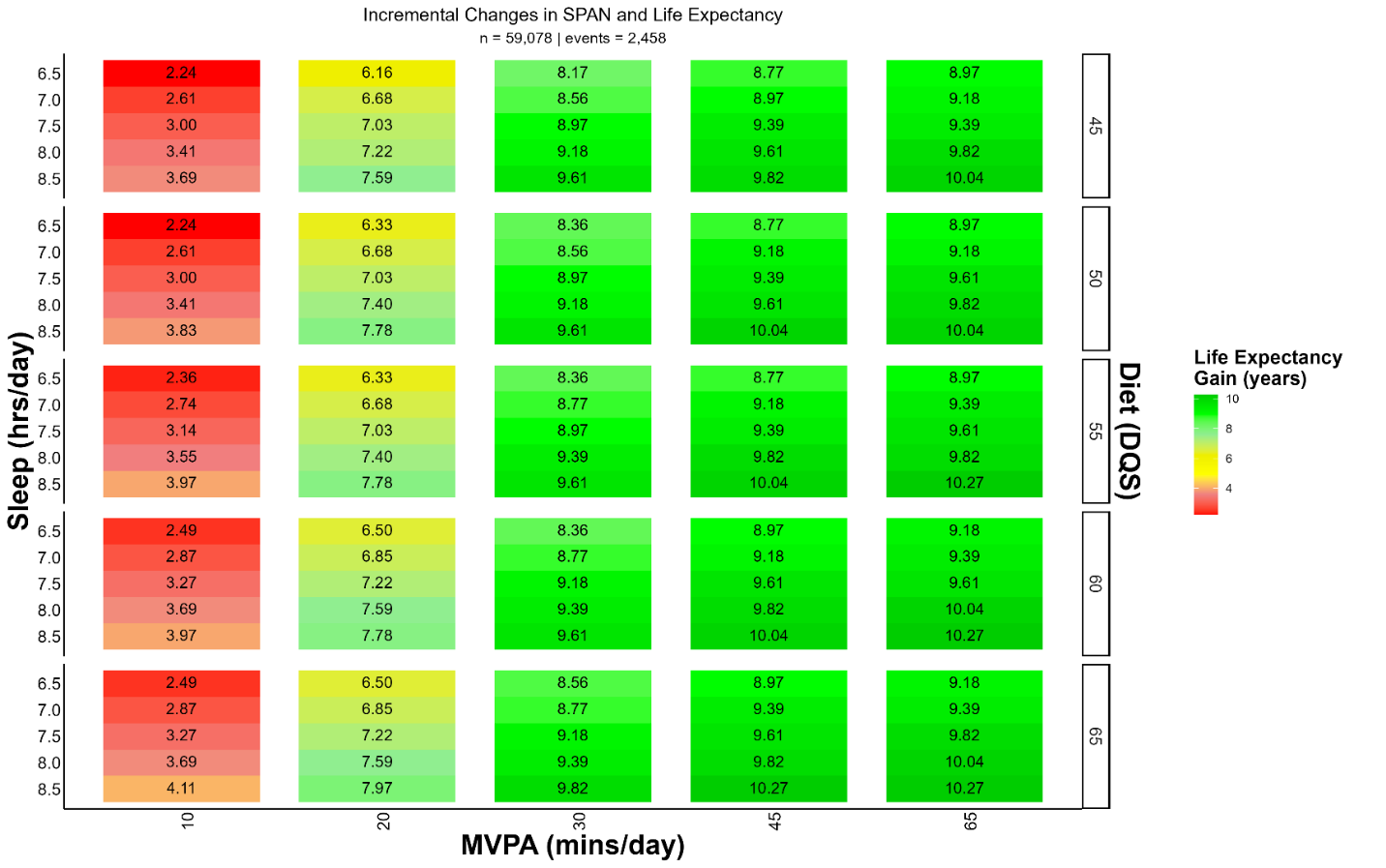


**Supplementary Figure 2.** Years of lifespan gained with concurrent variations in sleep, physical activity, and nutrition (n = 59,078; all-cause mortality events = 2,458)

**Legend:** The correlogram displays changes in sleep (hrs/day), physical activity (moderate to vigorous intensity (MVPA) minutes/day), and nutrition (Dietary Quality Score (DQS)) and corresponding years of life expectancy (lifespan) gained with the reference being the 5th percentile of sleep (5.5 hours/day), physical activity (7.3 minutes/day), and nutrition (36.9 DQS). Sleep, physical activity, and nutrition are included as independent terms in the model to allow for more granular predictions. Life expectancy was estimated using life table models, with predictions based on hazard ratio-adjusted mortality rates for all-cause mortality. The all-cause mortality is adjusted for age, sex, ethnicity, smoking, education, Townsend deprivation index, alcohol, discretionary screen time (time spent watching TV or using the computer outside of work), light intensity physical activity, medication (blood pressure, insulin, and cholesterol), previous diagnosis of major cardiovascular disease (defined as a disease of the circulatory system, arteries, and lymph, excluding hypertension), previous diagnosis of cancer, and familial history of cardiovascular disease and cancer.


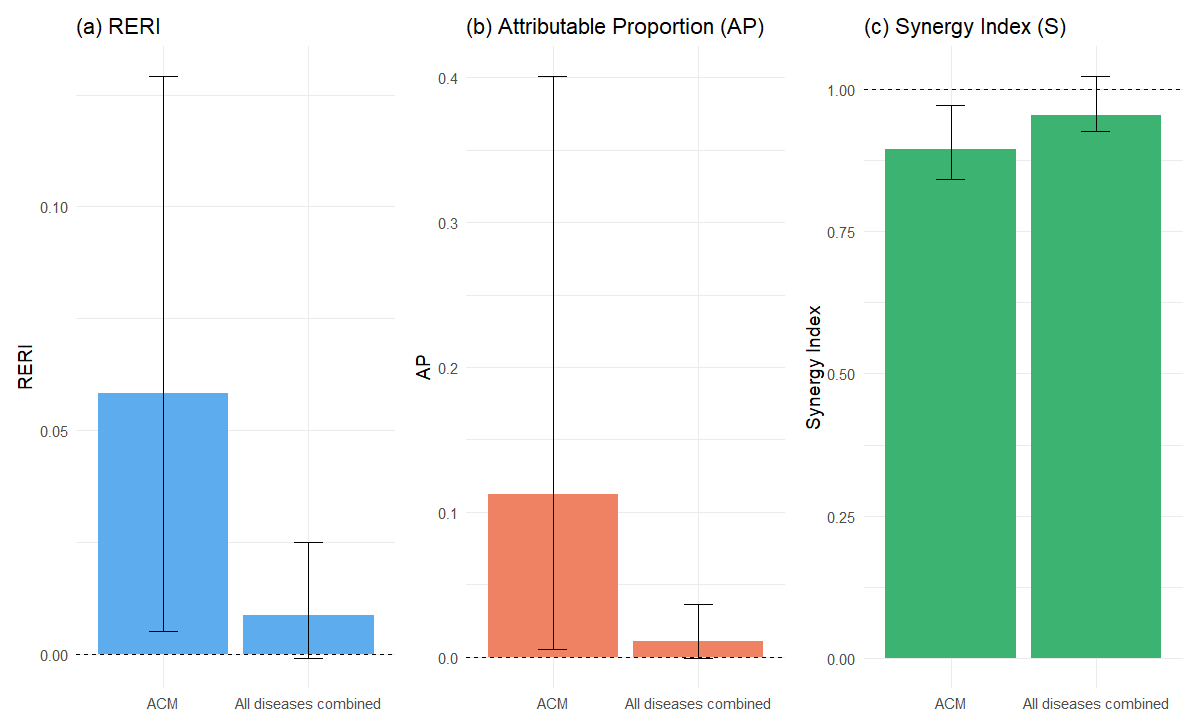


**Supplementary Figure 3**. Synergistic relationship between sleep, physical activity, and nutrition in relation to mortality and morbidity

**Legend**: The figure above shows the individual and interactive model terms of sleep, physical activity, and nutrition for all-cause mortality (ACM); type II diabetes (T2D); cardiovascular disease (CVD), and chronic obstructive pulmonary disease (COPD). To test for interactive and synergistic effects, we calculated the relative excess risk due to interaction (RERI), attributable proportion due to interaction (AP), and the synergistic effects index (S)^1^. These tests provide insight into the contribution of synergistic interactions between exposures, where an RERI or AP of 0 and an S value of 1 denote no interaction effect.


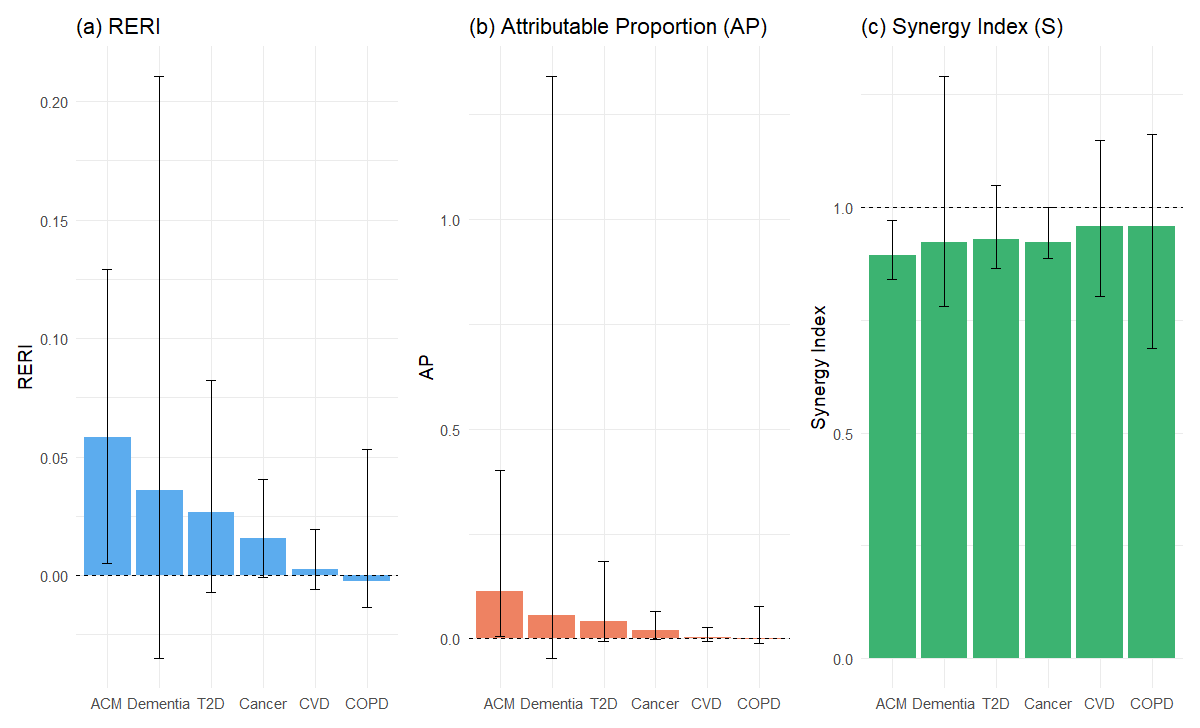


**Supplementary Figure 4**. Synergistic relationship between sleep, physical activity, and nutrition in relation to mortality and morbidity by individual diseases

**Legend**: The figure above shows the individual and interactive model terms of sleep, physical activity, and nutrition for all-cause mortality (ACM); type II diabetes (T2D); cardiovascular disease (CVD), and chronic obstructive pulmonary disease (COPD). To test for interactive and synergistic effects, we calculated the relative excess risk due to interaction (RERI), attributable proportion due to interaction (AP), and the synergistic effects index (S)^1^. These tests provide insight into the contribution of synergistic interactions between exposures, where an RERI or AP of 0 and an S value of 1 denote no interaction effect.

**
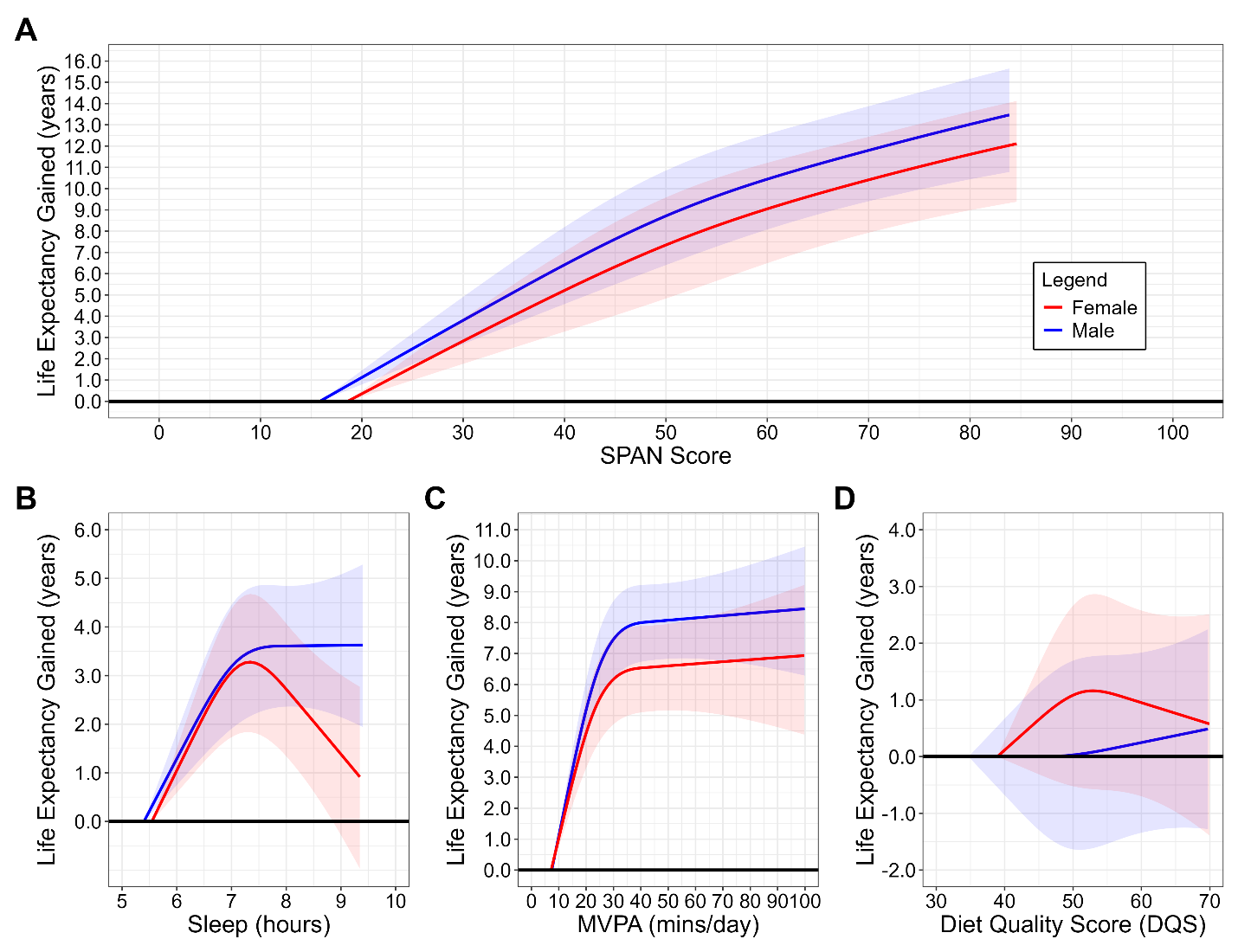
**

**Supplementary Figure 5.** Multivariable-adjusted dose-response association between A) SPAN score B) sleep C) physical activity and D) nutrition lifespan in males (n = 26,810; all-cause mortality events = 1,520) and females (n = 32,268; all-cause mortality events = 938)

**Legend:** Life expectancy was estimated using stratified sex-specific life table models, with predictions based on hazard ratio-adjusted mortality rates for the association between the composite sleep, physical activity, and nutrition (SPAN) and all-cause mortality. The all-cause mortality model is adjusted for age, ethnicity, smoking, education, Townsend deprivation index, alcohol, discretionary screen time (time spent watching TV or using the computer outside of work), light intensity physical activity, medication (blood pressure, insulin, and cholesterol), previous diagnosis of major cardiovascular disease (defined as disease of the circulatory system, arteries, and lymph, excluding hypertension), previous diagnosis of cancer, and familial history of cardiovascular disease and cancer. The SPAN score is comprised of sleep (hours/day), physical activity (moderate to vigorous intensity – MVPA, minutes/day), and nutrition (Dietary Quality Score, DQS) were combined as continuous variables, each weighted equally, with scores ranging from 0 to 100. Higher scores indicated a more beneficial combined SPAN value and the referent point used was the 5^th^ percentile for each sex. The weighting of each exposure within the SPAN score was determined based on the theoretically optimal levels identified from the dose-response relationship with all-cause mortality. In figures B-D, the individual dose-response relationship between sleep, physical activity, nutrition and life expectancy was examined using the sex-specific 5^th^ percentile for each exposure as the referent point.


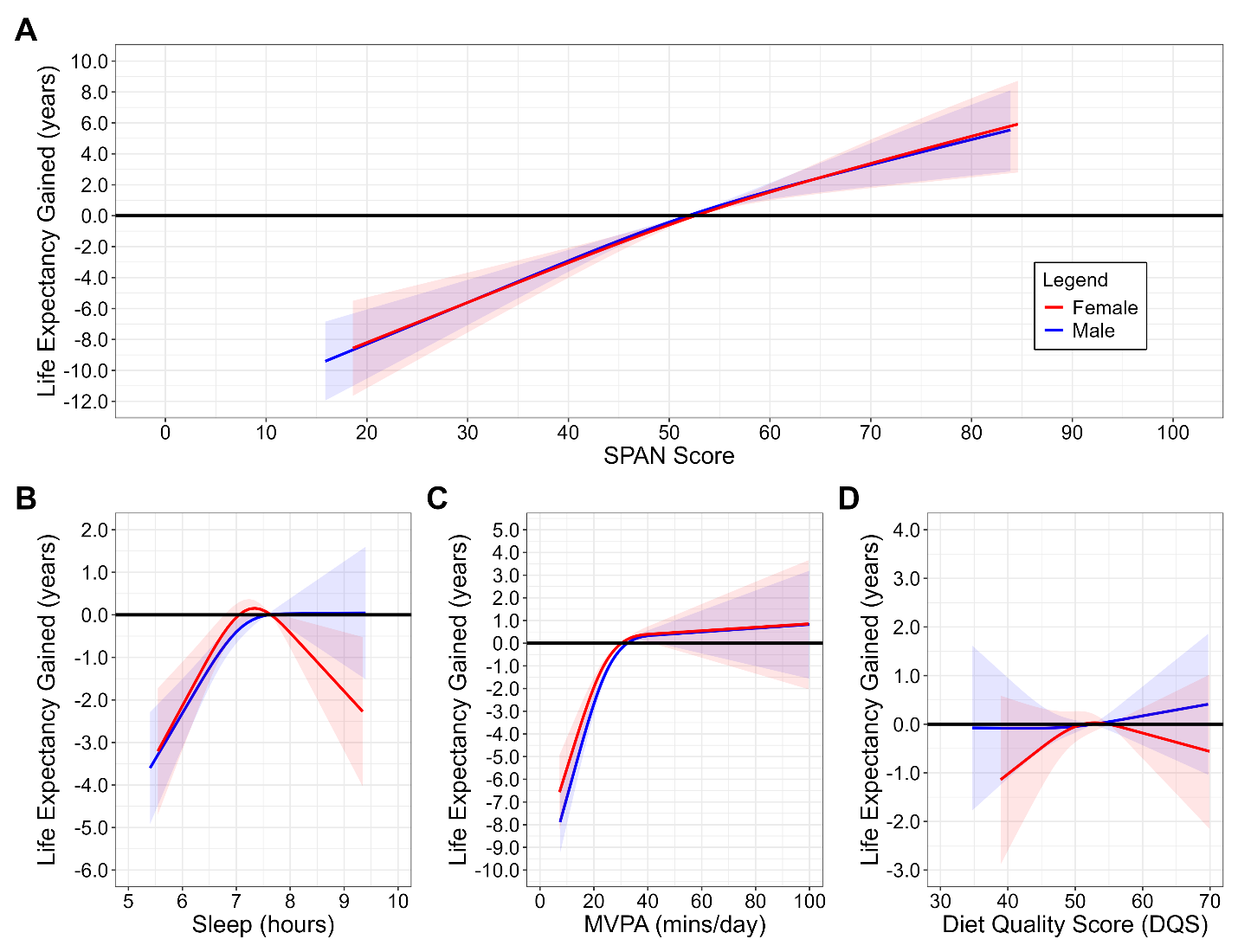


**Supplementary Figure 6.** Multivariable-adjusted dose-response association between B) sleep C) physical activity and D) nutrition with lifespan in males (n = 26,810; all-cause mortality events = 1,520) and females (n = 32,268; all-cause mortality events = 938) using alternative referent point

**Legend:** Life expectancy (lifespan) was estimated using stratified sex-specific life table models, with predictions based on hazard ratio-adjusted mortality rates for the association between the composite sleep, physical activity, and nutrition (SPAN) and all-cause mortality. The all-cause mortality model is adjusted for age, ethnicity, smoking, education, Townsend deprivation index, alcohol, discretionary screen time (time spent watching TV or using the computer outside of work), light intensity physical activity, medication (blood pressure, insulin, and cholesterol), previous diagnosis of major cardiovascular disease (defined as disease of the circulatory system, arteries, and lymph, excluding hypertension), previous diagnosis of cancer, and familial history of cardiovascular disease and cancer. The SPAN score is comprised of sleep (hours/day), physical activity (moderate to vigorous intensity – MVPA, minutes/day), and nutrition (Dietary Quality Score, DQS) were combined as continuous variables, each weighted equally, with scores ranging from 0 to 100. Higher scores indicated a more beneficial combined SPAN value and the referent point used was the median value for each sex. The weighting of each exposure within the SPAN score was determined based on the theoretically optimal levels identified from the dose-response relationship with all-cause mortality. In figures B-D, the individual dose-response relationship between sleep, physical activity, nutrition and life expectancy was examined using the sex-specific median value for each exposure as the referent point.

**
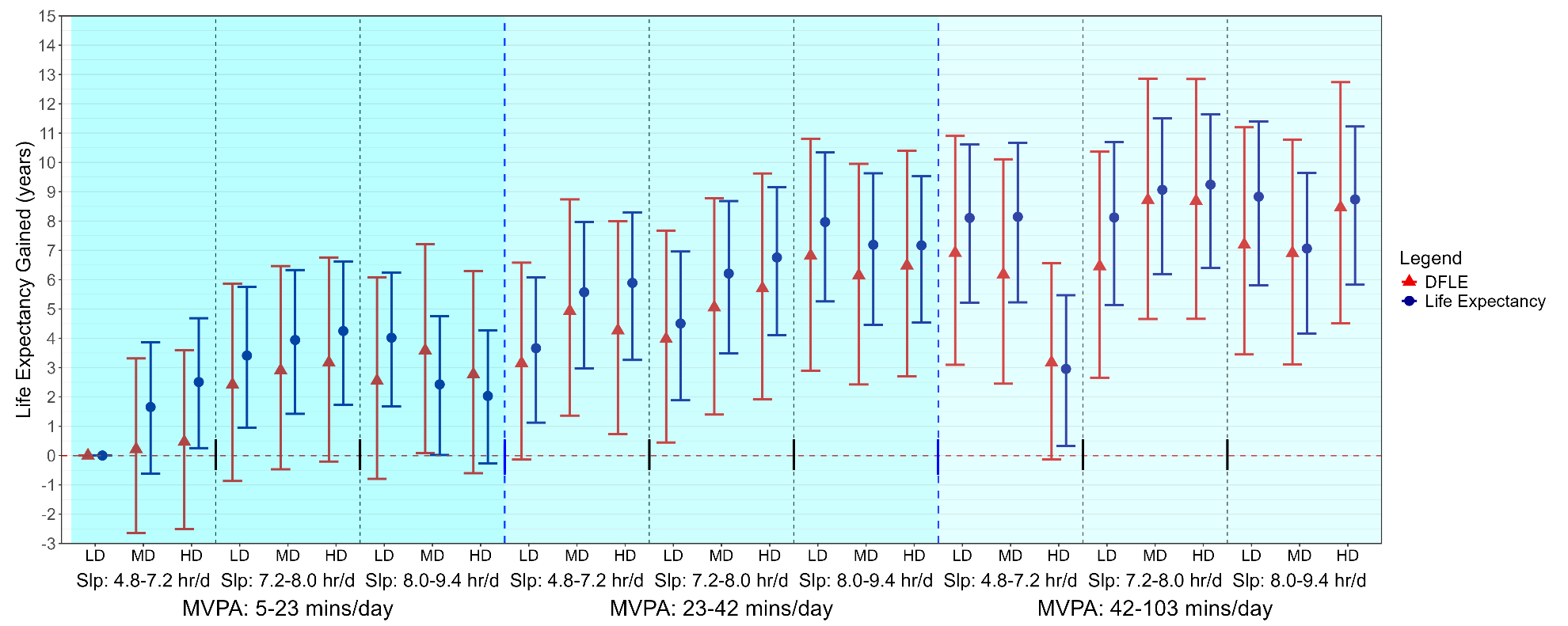
**

**Supplemental Figure 7:** Multivariable-adjusted associations of combined sleep, physical activity, and nutrition with lifespan and healthspan expectancy following exclusion of individuals with poor health (n = 51,164; events = 1,887)

**Legend**: Forest plot shows the sleep, physical activity, and nutrition (SPAN) associations with life expectancy (lifespan) and disease free life expectancy (DFLE; healthspan) after removing those with poor health status including low BMI (<18.5), current smokers, self-reported poor health, and those with a frailty index score of >3. Life expectancy was estimated using life table models, with predictions based on hazard ratio-adjusted mortality rates for all-cause mortality associated with each SPAN category. Disease-free life expectancy was calculated as the expected lifespan free from cardiovascular disease, cancer, type II diabetes, chronic obstructive pulmonary disease (COPD), or dementia. DFLE incorporated a life table approach that included age-specific incidence rates for each condition. The all-cause mortality is adjusted for age, sex, ethnicity, smoking, education, Townsend deprivation index, alcohol, discretionary screen time (time spent watching TV or using the computer outside of work), light intensity physical activity, medication (blood pressure, insulin, and cholesterol), previous diagnosis of major cardiovascular disease (defined as disease of the circulatory system, arteries, and lymph, excluding hypertension), previous diagnosis of cancer, and familial history of cardiovascular disease and cancer. Sleep (hours/day), physical activity (moderate to vigorous intensity – MVPA- minutes/day), and nutrition (Dietary Quality Score, DQS) were included in the model as a joint term. Dashed blue lines separate tertiles MVPA and dashed black lines separate tertiles of sleep. Sleep (Slp); Low Diet Quality (LD); Medium Diet Quality (MD); High Diet Quality (HD).

**
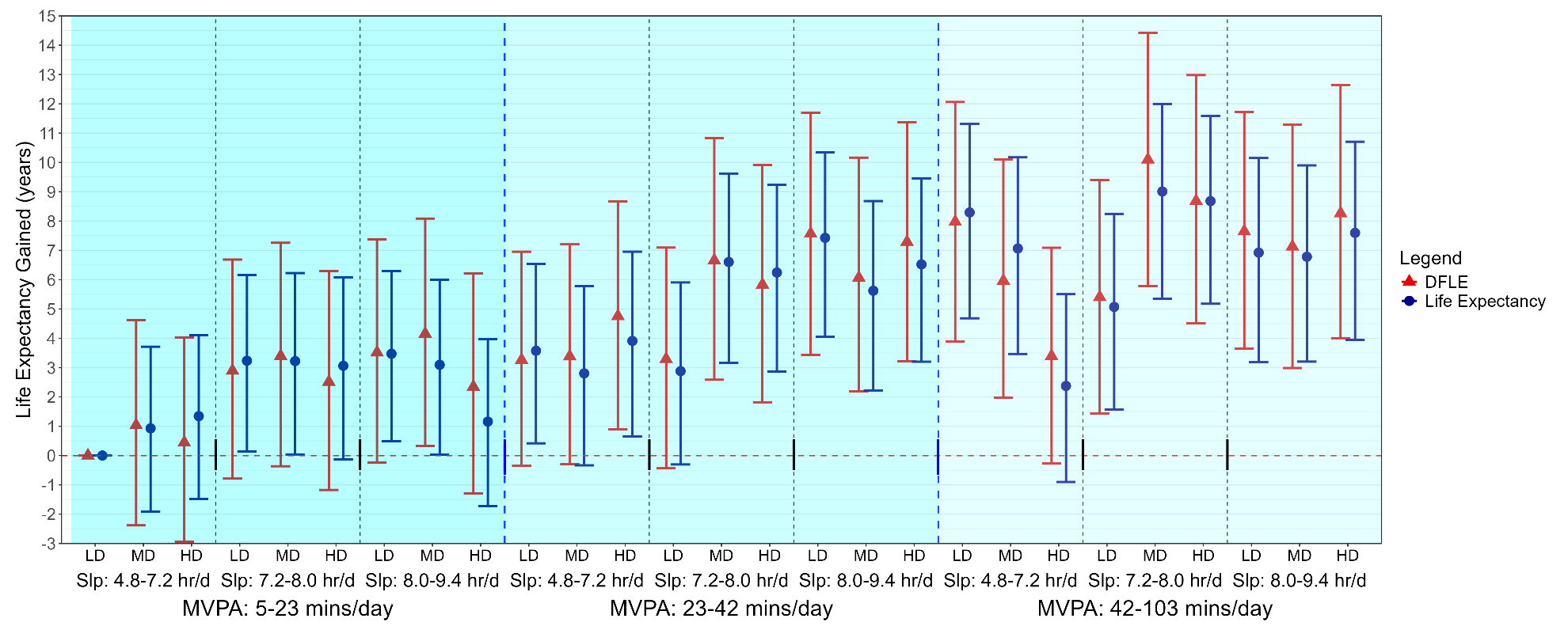
**

**Supplementary Figure 8:** Multivariable-adjusted associations of combined sleep, physical activity, and nutrition with lifespan and healthspan excluding individuals with baseline chronic conditions (n = 51,166; events = 1,888)

**Legend**: Forest plot shows the sleep, physical activity, and nutrition (SPAN) associations with life expectancy (lifespan) and disease free life expectancy (DFLE; healthspan) after removing those with baseline chronic conditions including cardiovascular disease (CVD), cancer, chronic obstructive pulmonary disease (COPD), dementia, or type II diabetes. Model is adjusted for age, sex, ethnicity, smoking, education, Townsend deprivation index, alcohol, discretionary screen time (time spent watching TV or using the computer outside of work), light intensity physical activity, medication (blood pressure, insulin, and cholesterol), and familial history of CVD and cancer. Sleep (hours/day), physical activity (moderate to vigorous intensity (MVPA) minutes/day), and nutrition (Dietary Quality Score (DQS)) were included in the model as a joint term. The specific ranges for each exposure included sleep duration as 5.0-7.2 hours/day (low), 7.2-8.0 hours/day (medium), and 8.0-9.4 hours/day (high); MVPA measurements as 6-23 minutes/day (low), 23-42 minutes/day (medium), and 43-104 minutes/day (high); and diet quality using the DQS as 34.0-50.0 (low), 50.0-57.5 (medium), and 57.5-72.5 (high). The lowest tertiles for all three exposures (sleep, MVPA and DQS) was the referent group. Dashed blue lines separate tertiles MVPA and dashed black lines separate tertiles of sleep. Sleep (Slp); Low Diet Quality (LD); Medium Diet Quality (MD); High Diet Quality (HD).

**
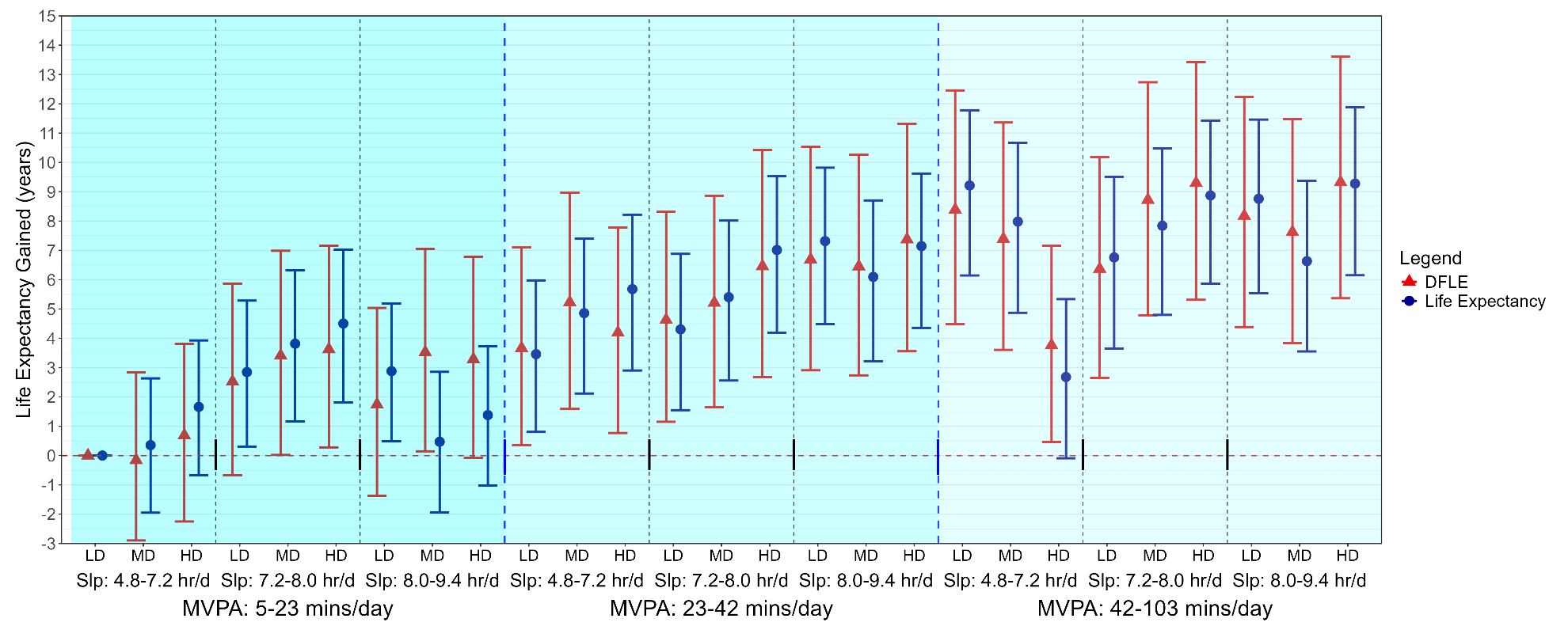
**

**Supplementary Figure 9:** Multivariable-adjusted associations of combined sleep, physical activity, and nutrition with lifespan and healthspan excluding individuals with an event in the first 3 years of follow-up (n = 58,610; events = 1,990)

**Legend**: Forest plot shows the sleep, physical activity, and nutrition (SPAN) associations with life expectancy (lifespan) and disease free life expectancy (DFLE; healthspan) after removing those with a mortality event in the first three years of follow-up. Model is adjusted for age, sex, ethnicity, smoking, education, Townsend deprivation index, alcohol, discretionary screen time (time spent watching TV or using the computer outside of work), light intensity physical activity, medication (blood pressure, insulin, and cholesterol), and familial history of cardiovascular disease and cancer. Sleep (hours/day), physical activity (moderate to vigorous intensity (MVPA) minutes/day), and nutrition (Dietary Quality Score (DQS)) were included in the model as a joint term. The specific ranges for each exposure included sleep duration as 5.0-7.2 hours/day (low), 7.2-8.0 hours/day (medium), and 8.0-9.4 hours/day (high); MVPA measurements as 6-23 minutes/day (low), 23-42 minutes/day (medium), and 43-104 minutes/day (high); and diet quality using the DQS as 34.0-50.0 (low), 50.0-57.5 (medium), and 57.5-72.5 (high). The lowest tertiles for all three exposures (sleep, MVPA and DQS) was the referent group. Dashed blue lines separate tertiles MVPA and dashed black lines separate tertiles of sleep. Sleep (Slp); Low Diet Quality (LD); Medium Diet Quality (MD); High Diet Quality (HD).

**
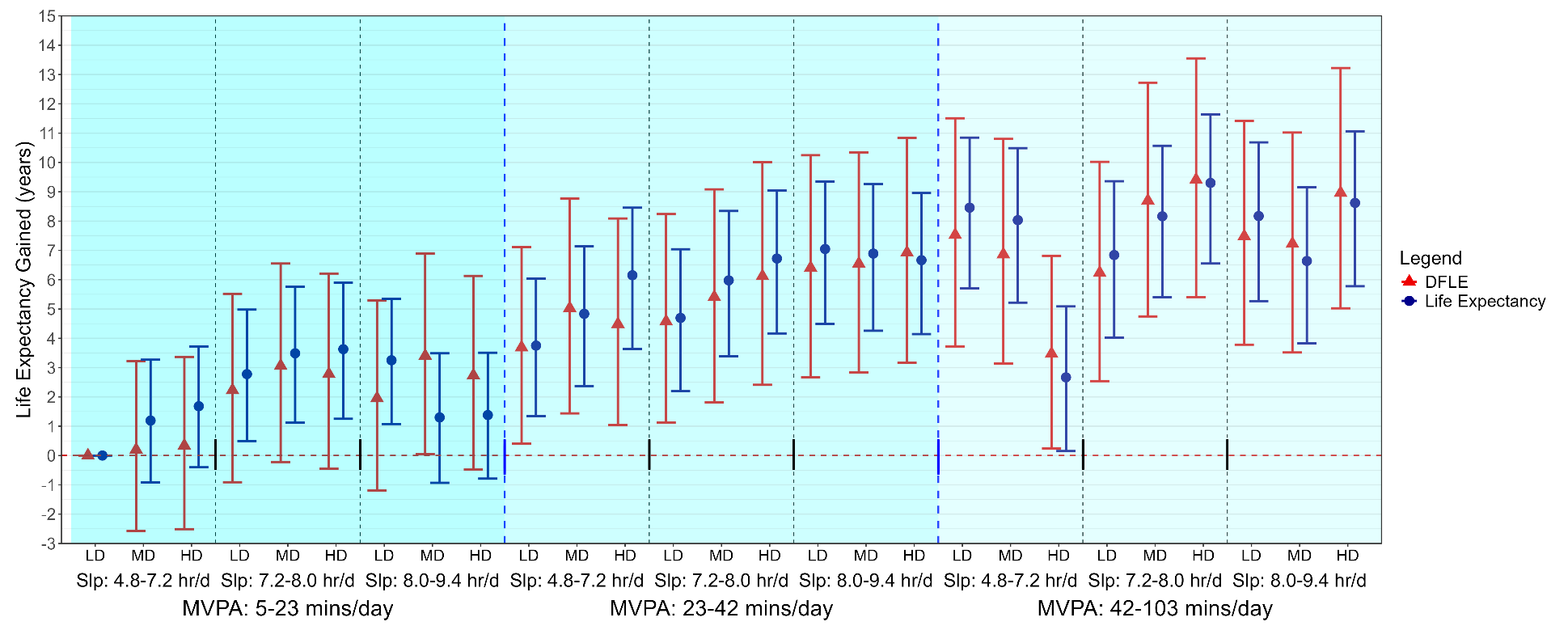
**

**Supplementary Figure 10**: Multivariable-adjusted associations of combined sleep, physical activity, and nutrition with lifespan and healthspan adjusted for BMI (n = 58,363; events = 2,405)

**Legend**: Life expectancy (lifespan) was estimated using life table models, with predictions derived from hazard ratio-adjusted mortality rates derived from the association between each mutually exclusive sleep, physical activity, and nutrition (SPAN) category and all-cause mortality. Disease-free life expectancy (DFLE; healthspan) was calculated as the expected lifespan free from cardiovascular disease, cancer, type II diabetes, chronic obstructive pulmonary disease (COPD), or dementia. DFLE incorporated a life table approach that included age-specific incidence rates for each condition. Model is adjusted for age, sex, ethnicity, smoking, education, Townsend deprivation index, alcohol, discretionary screen time (time spent watching TV or using the computer outside of work), light intensity physical activity, medication (blood pressure, insulin, and cholesterol), previous diagnosis of major cardiovascular disease (defined as disease of the circulatory system, arteries, and lymph, excluding hypertension), previous diagnosis of cancer, familial history of cardiovascular disease and cancer, and BMI. Sleep (hours/day), physical activity (moderate to vigorous intensity (MVPA) minutes/day), and nutrition (Dietary Quality Score (DQS)) were included in the model as a joint term. The specific ranges for each exposure included sleep duration as 4.8-7.2 hours/day (low), 7.2-8.0 hours/day (medium), and 8.0-9.4 hours/day (high); MVPA measurements as 5-23 minutes/day (low), 23-42 minutes/day (medium), and 42-103 minutes/day (high); and diet quality using the DQS as 32.5-50.0 (low), 50.0-57.5 (medium), and 57.5-72.5 (high). The lowest tertiles for all three exposures (sleep, MVPA and DQS) were considered the reference group. Dashed blue lines separate tertiles MVPA and dashed black lines separate tertiles of sleep. Sleep (Slp); Low Diet Quality (LD); Medium Diet Quality (MD); High Diet Quality (HD).

**
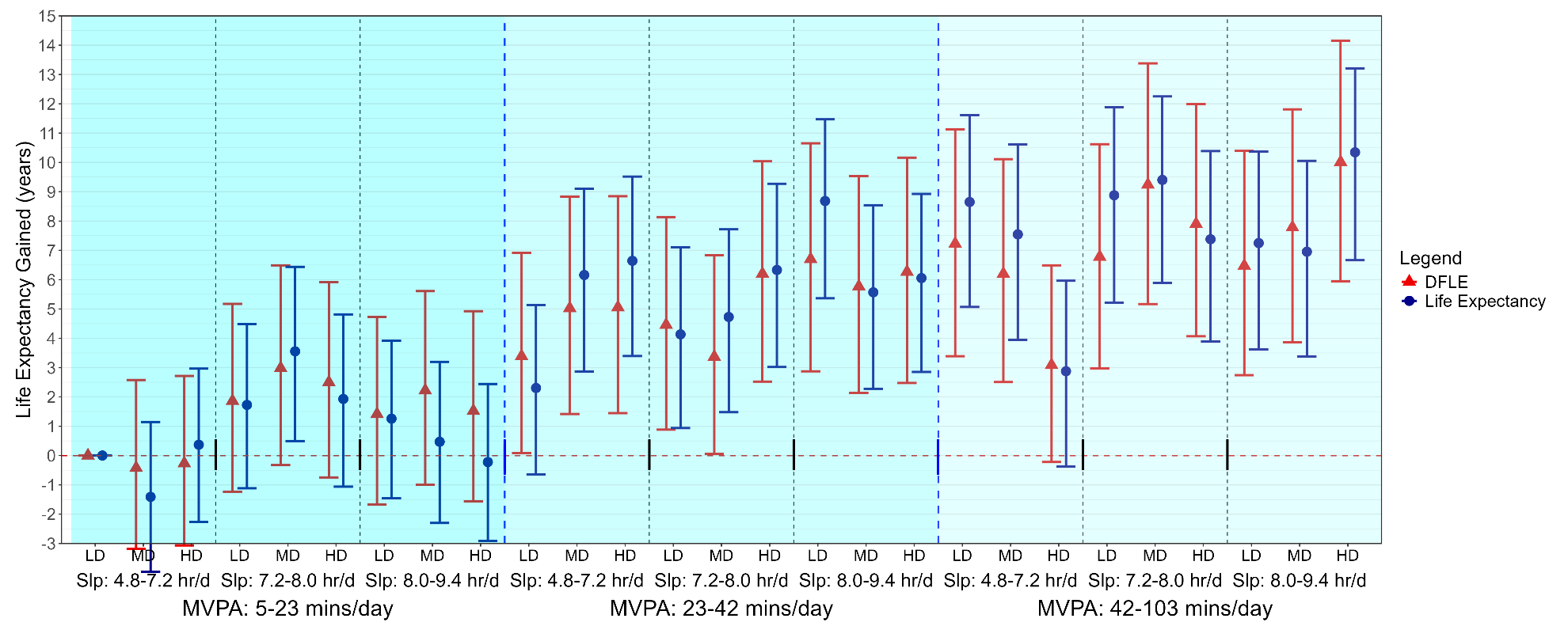
**

**Supplementary Figure 11:** Multivariable-adjusted associations of combined sleep, physical activity, and nutrition with lifespan and healthspan adjusted for sleep characteristics (n = 37,475; events = 1,506)

**Legend**: Life expectancy (lifespan) was estimated using life table models, with predictions derived from hazard ratio-adjusted mortality rates derived from the association between each mutually exclusive sleep, physical activity, and nutrition (SPAN) category and all-cause mortality. Disease-free life expectancy (DFLE; healthspan) was calculated as the expected lifespan free from cardiovascular disease, cancer, type II diabetes, chronic obstructive pulmonary disease (COPD), or dementia. DFLE incorporated a life table approach that included age-specific incidence rates for each condition. Model is adjusted for age, sex, ethnicity, smoking, education, Townsend deprivation index, alcohol, discretionary screen time (time spent watching TV or using the computer outside of work), light intensity physical activity, medication (blood pressure, insulin, and cholesterol), previous diagnosis of major cardiovascular disease (defined as disease of the circulatory system, arteries, and lymph, excluding hypertension), previous diagnosis of cancer, familial history of cardiovascular disease and cancer, insomnia, snoring, chronotype (morning/evening person), and daytime sleepiness. Sleep (hours/day), physical activity (moderate to vigorous intensity (MVPA) minutes/day), and nutrition (Dietary Quality Score (DQS)) were included in the model as a joint term. The specific ranges for each exposure included sleep duration as 4.8-7.2 hours/day (low), 7.2-8.0 hours/day (medium), and 8.0-9.4 hours/day (high); MVPA measurements as 5-23 minutes/day (low), 23-42 minutes/day (medium), and 42-103 minutes/day (high); and diet quality using the DQS as 32.5-50.0 (low), 50.0-57.5 (medium), and 57.5-72.5 (high). The lowest tertiles for all three exposures (sleep, MVPA and DQS) were considered the reference group. Dashed blue lines separate tertiles MVPA and dashed black lines separate tertiles of sleep. Sleep (Slp); Low Diet Quality (LD); Medium Diet Quality (MD); High Diet Quality (HD).


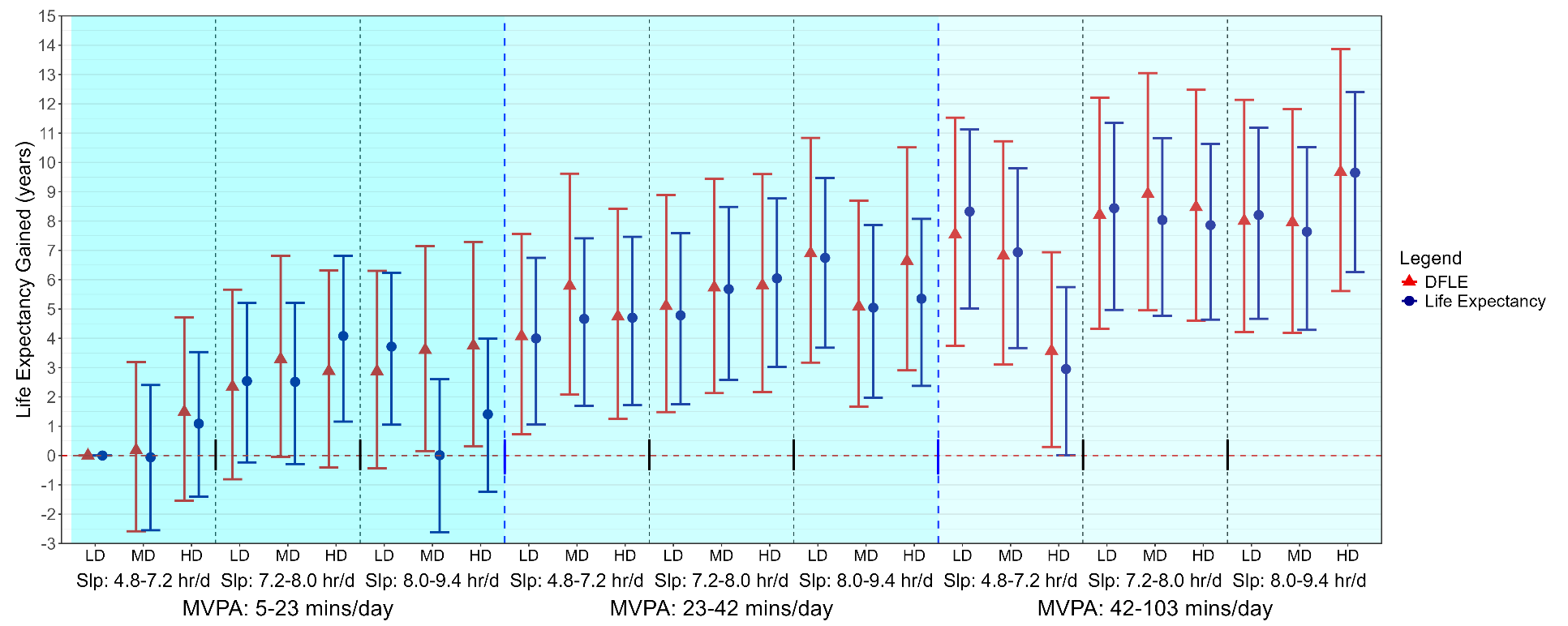


**Supplementary Figure 12:** Multivariable-adjusted associations of combined sleep, physical activity, and nutrition with adjusted lifespan and healthspan using the proportion of ultra-processed food (n = 43,694; events = 1,758)

**Legend**: Life expectancy (lifespan) was estimated using life table models, with predictions derived from hazard ratio-adjusted mortality rates derived from the association between each mutually exclusive sleep, physical activity, and nutrition (SPAN) category and all-cause mortality. Disease-free life expectancy (DFLE; healthspan) was calculated as the expected lifespan free from cardiovascular disease, cancer, type II diabetes, chronic obstructive pulmonary disease (COPD), or dementia. DFLE incorporated a life table approach that included age-specific incidence rates for each condition. Model is adjusted for age, sex, ethnicity, smoking, education, Townsend deprivation index, alcohol, discretionary screen time (time spent watching TV or using the computer outside of work), light intensity physical activity, medication (blood pressure, insulin, and cholesterol), previous diagnosis of major cardiovascular disease (defined as disease of the circulatory system, arteries, and lymph, excluding hypertension), previous diagnosis of cancer, familial history of cardiovascular disease and cancer. From 2009-2012, dietary data was also collected using 1-4 separate 24-hour dietary recall for a subgroup of participants (n = 211,031)1. Diet quality was defined as the percentage of dietary ultra-processed food where higher diet quality had a lower proportion of ultra-processed food in the diet. Sleep (hours/day), physical activity (moderate to vigorous intensity (MVPA) minutes/day), and nutrition (ultra-processed food intake, % of total diet by weight) were included in the model as a joint term. The specific ranges for each exposure included sleep duration as 4.8-7.2 hours/day (low), 7.2-8.0 hours/day (medium), and 8.0-9.4 hours/day (high); MVPA measurements as 5-23 minutes/day (low), 23-42 minutes/day (medium), and 42-103 minutes/day (high); and diet quality using the proportion of ultra-processed food as 21.5-100.0 (low), 13.2-21.5 (medium), and 0.0-13.2% (high). The lowest tertiles for all three exposures (sleep, MVPA and DQS) were considered the reference group. Dashed blue lines separate tertiles MVPA and dashed black lines separate tertiles of sleep. Sleep (Slp); Low Diet Quality (LD); Medium Diet Quality (MD); High Diet Quality (HD).

**
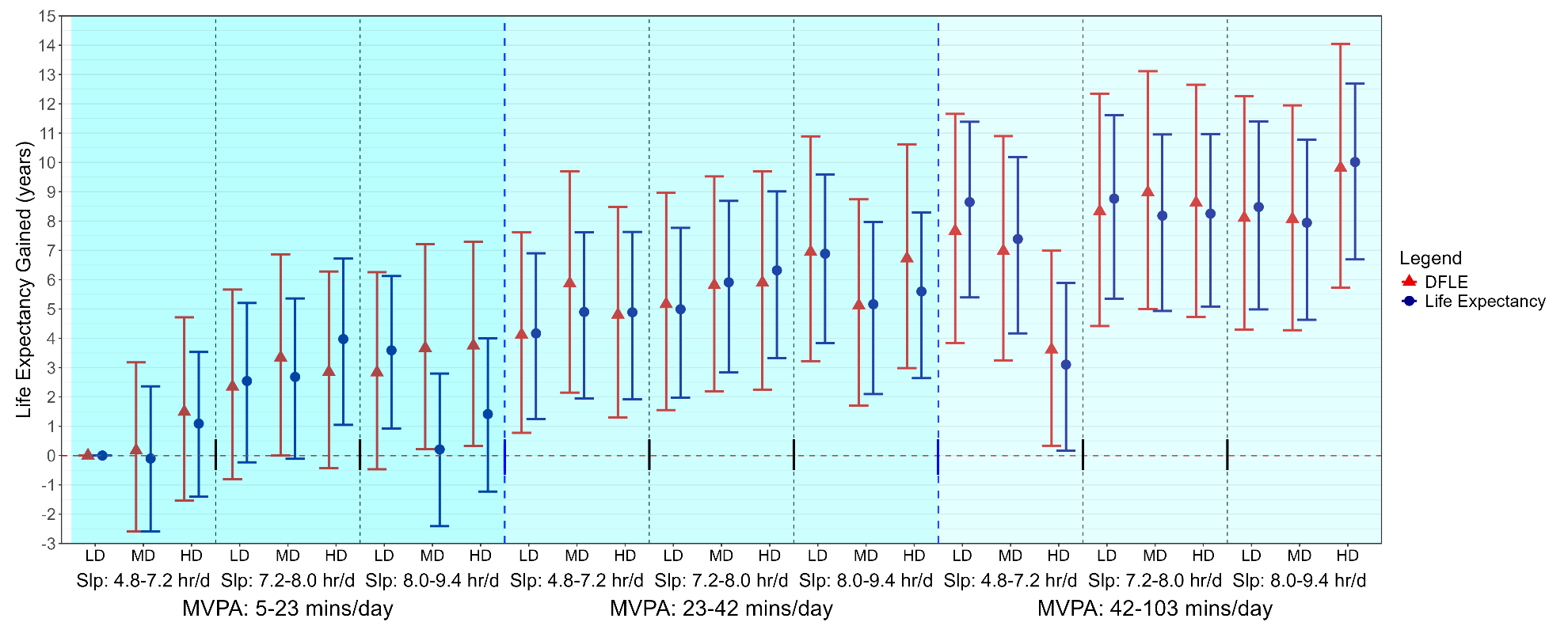
Supplementary Figure 13:** Multivariable-adjusted associations of combined sleep, physical activity, and nutrition with lifespan and healthspan adjusted for total energy intake (n = 42,990; 1,758 events)

**Legend**: Life expectancy (lifespan) was estimated using life table models, with predictions derived from hazard ratio-adjusted mortality rates derived from the association between each mutually exclusive sleep, physical activity, and nutrition (SPAN) category and all-cause mortality. Disease-free life expectancy (DFLE; healthspan) was calculated as the expected lifespan free from cardiovascular disease, cancer, type II diabetes, chronic obstructive pulmonary disease (COPD), or dementia. DFLE incorporated a life table approach that included age-specific incidence rates for each condition. Model is adjusted for age, sex, ethnicity, smoking, education, Townsend deprivation index, alcohol, discretionary screen time (time spent watching TV or using the computer outside of work), light intensity physical activity, medication (blood pressure, insulin, and cholesterol), previous diagnosis of major cardiovascular disease (defined as disease of the circulatory system, arteries, and lymph, excluding hypertension), previous diagnosis of cancer, familial history of cardiovascular disease and cancer, and total energy intake. From 2009-2012, dietary data was also collected using 1-4 separate 24-hour dietary recall for a subgroup of participants (n = 211,031)1. Energy intake outliers (<800 or >4200 kcal/ per day) for men and (<600 or >3500 kcal per day) for women were also excluded from this sample^2^. Sleep (hours/day), physical activity (moderate to vigorous intensity (MVPA) minutes/day), and nutrition (Dietary Quality Score (DQS)) were included in the model as a joint term. The specific ranges for each exposure included sleep duration as 4.8-7.2 hours/day (low), 7.2-8.0 hours/day (medium), and 8.0-9.4 hours/day (high); MVPA measurements as 5-23 minutes/day (low), 23-42 minutes/day (medium), and 42-103 minutes/day (high); and diet quality using the DQS as 32.5-50.0 (low), 50.0-57.5 (medium), and 57.5-72.5 (high). The lowest tertiles for all three exposures (sleep, MVPA and DQS) were considered the reference group. Dashed blue lines separate tertiles MVPA and dashed black lines separate tertiles of sleep. Sleep (Slp); Low Diet Quality (LD); Medium Diet Quality (MD); High Diet Quality (HD).


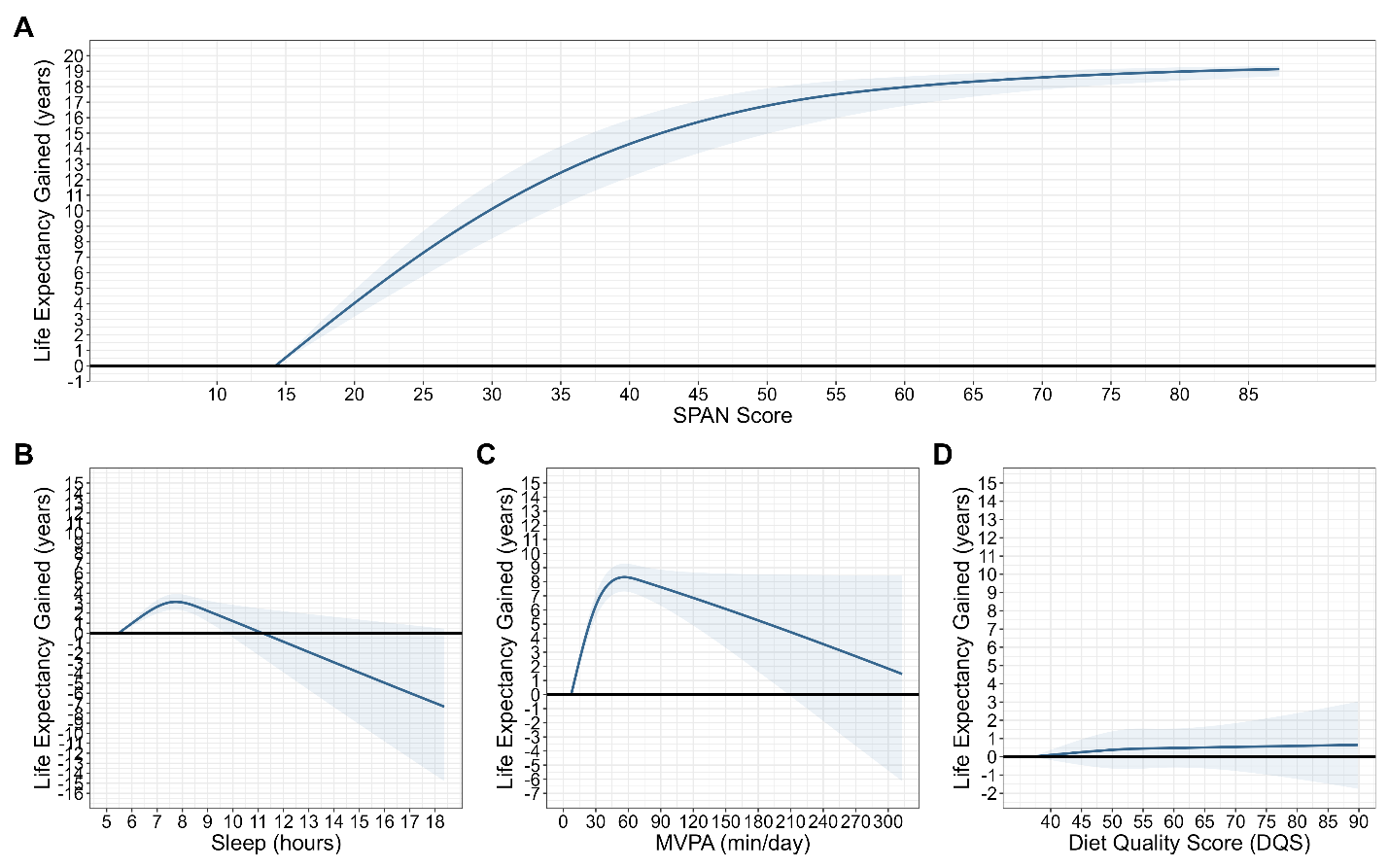


**Supplementary Figure 14**: Multivariable-adjusted associations of combined sleep, physical activity, and nutrition with lifespan and healthspan with no winsorisation (n = 59,078; all-cause mortality events = 2,458)

**Legend:** This analysis includes the full raw (non-winsorised) data for sleep, physical activity, nutrition (SPAN) in the composite SPAN score. Life expectancy (lifespan) was estimated using life table models, with predictions derived from hazard ratio-adjusted mortality rates for all-cause mortality associated with each category. Disease-free life expectancy (DFLE; healthspan) was calculated as the expected lifespan free from cardiovascular disease, cancer, type II diabetes, chronic obstructive pulmonary disease, or dementia. DFLE incorporated a life table approach that included age-specific incidence rates for each condition. The all-cause mortality model is adjusted for age, sex, ethnicity, smoking, education, Townsend deprivation index, alcohol, discretionary screen time (time spent watching TV or using the computer outside of work), light intensity physical activity, medication (blood pressure, insulin, and cholesterol), previous diagnosis of major cardiovascular disease (defined as disease of the circulatory system, arteries, and lymph, excluding hypertension), previous diagnosis of cancer, and familial history of cardiovascular disease and cancer.The SPAN score is comprised of sleep (hours/day), physical activity (moderate to vigorous intensity – MVPA, minutes/day), and nutrition (Dietary Quality Score, DQS) were combined as continuous variables, each weighted equally, with scores ranging from 0 to 100. Higher scores indicated a more beneficial combined SPAN value, and the referent point used was the 5^th^ percentile of the composite score. The weighting of each exposure within the SPAN score was determined based on the theoretically optimal levels identified from the dose-response relationship with all-cause mortality. In figures B-D, the individual dose-response relationship between sleep, physical activity, nutrition and life expectancy was examined using the 5^th^ percentile for each exposure as the referent point.


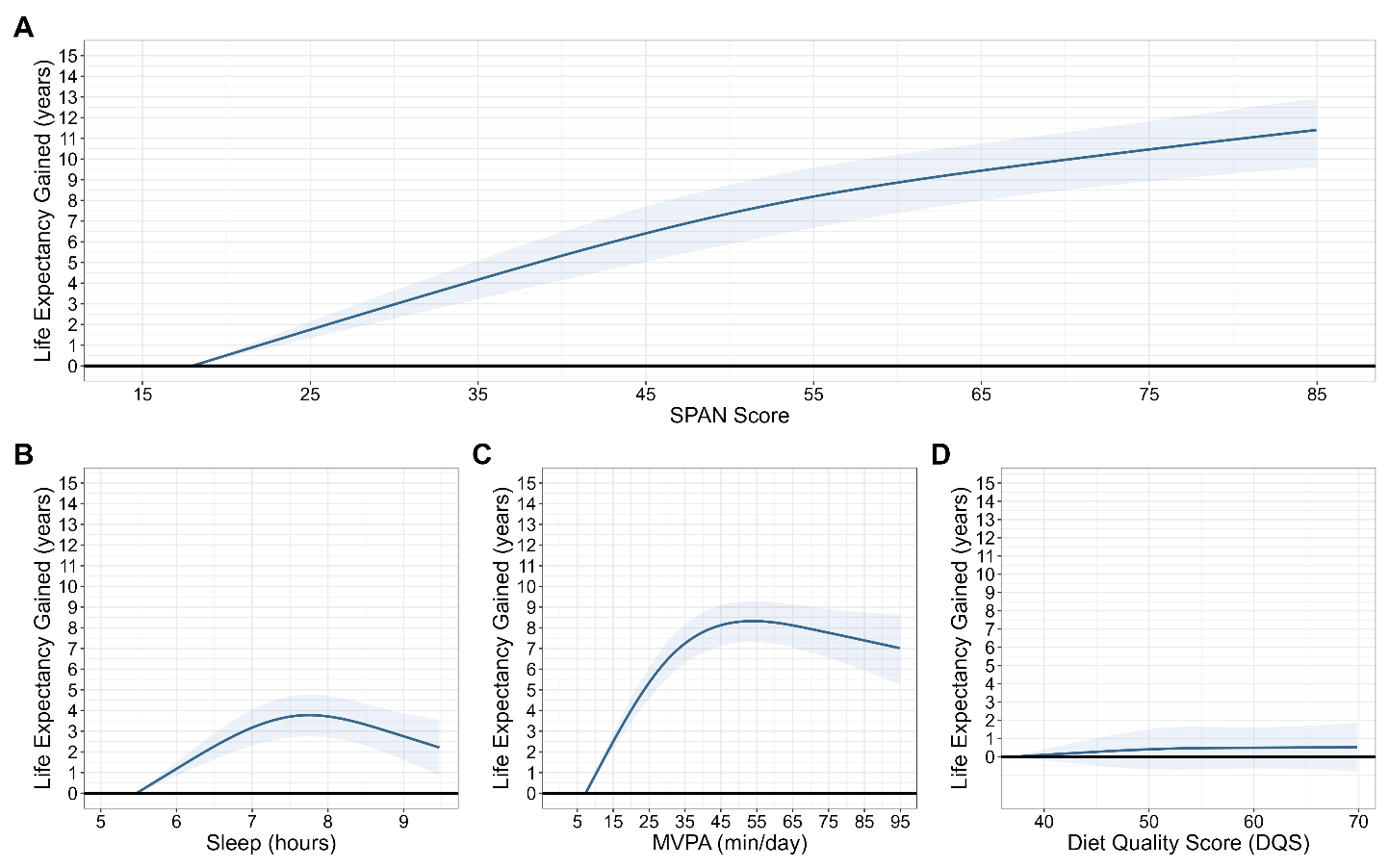


**Supplementary Figure 15**: Multivariable-adjusted associations of combined sleep, physical activity, and nutrition with lifespan and healthspan using Tukey’s Fences as an alternative winsorisation threshold (n = 59,078; all-cause mortality events = 2,458)

**Legend:** This analysis employs the Tukey’s method^3^ for determining the appropriate winsorisation thresholds for sleep, physical activity, and nutrition (SPAN) in the composite SPAN score. Tukey’s approach defines extreme values using the interquartile range, where observations below Q1 minus 1.5 × IQR or above Q3 plus 1.5 × IQR are treated as outliers and capped at these limits. Life expectancy (lifespan) was estimated using life table models, with predictions derived from hazard ratio-adjusted mortality rates for all-cause mortality associated with each category. Disease-free life expectancy (DFLE; healthspan) was calculated as the expected lifespan free from cardiovascular disease, cancer, type II diabetes, chronic obstructive pulmonary disease, or dementia. DFLE incorporated a life table approach that included age-specific incidence rates for each condition. The all-cause mortality model is adjusted for age, sex, ethnicity, smoking, education, Townsend deprivation index, alcohol, discretionary screen time (time spent watching TV or using the computer outside of work), light intensity physical activity, medication (blood pressure, insulin, and cholesterol), previous diagnosis of major cardiovascular disease (defined as disease of the circulatory system, arteries, and lymph, excluding hypertension), previous diagnosis of cancer, and familial history of cardiovascular disease and cancer. The SPAN score is comprised of sleep (hours/day), physical activity (moderate to vigorous intensity – MVPA, minutes/day), and nutrition (Dietary Quality Score, DQS) were combined as continuous variables, each weighted equally, with scores ranging from 0 to 100. Higher scores indicated a more beneficial combined SPAN value, and the referent point used was the 5^th^ percentile of the composite score. The weighting of each exposure within the SPAN score was determined based on the theoretically optimal levels identified from the dose-response relationship with all-cause mortality. In figures B-D, the individual dose-response relationship between sleep, physical activity, nutrition and life expectancy was examined using the 5^th^ percentile for each exposure as the referent point.


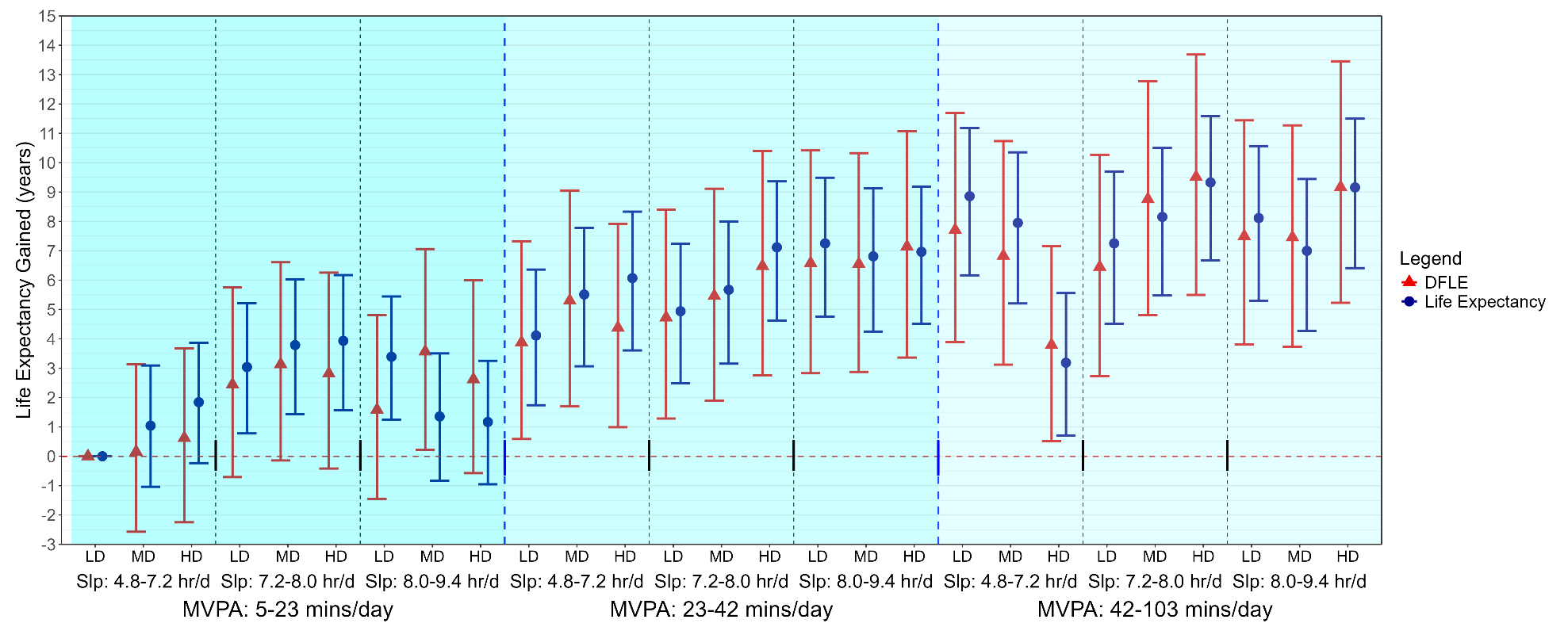


**Supplementary Figure 16:** Multivariable-adjusted associations of combined sleep, physical activity, and nutrition with lifespan and healthspan using an imputed data set for incomplete covariate data (n = 59,931; all-cause mortality events = 2,505)

**Legend:** This analysis employs multiple imputation by chained equations^4^ to account for missing covariates in the dataset and increase the amount of usable data in the core analytical sample. Five imputed datasets were generated with plausible values for missing covariates predicted from the distribution of the variables. Datasets were then combined following Rubin’s rules^4^ to generate pooled estimates. Life expectancy (lifespan) was estimated using life table models, with predictions derived from hazard ratio-adjusted mortality rates derived from the association between each mutually exclusive sleep, physical activity, and nutrition (SPAN) category and all-cause mortality. Disease-free life expectancy (DFLE; healthspan) was calculated as the expected lifespan free from cardiovascular disease, cancer, type II diabetes, chronic obstructive pulmonary disease, or dementia. DFLE incorporated a life table approach that included age-specific incidence rates for each condition. The all-cause mortality model is adjusted for age, sex, ethnicity, smoking, education, Townsend deprivation index, alcohol, discretionary screen time (time spent watching TV or using the computer outside of work), light intensity physical activity, medication (blood pressure, insulin, and cholesterol), previous diagnosis of major cardiovascular disease (defined as disease of the circulatory system, arteries, and lymph, excluding hypertension), previous diagnosis of cancer, and familial history of cardiovascular disease and cancer. The specific ranges for each exposure included sleep duration as 4.8-7.2 hours/day (low), 7.2-8.0 hours/day (medium), and 8.0-9.4 hours/day (high); MVPA time: 5-23 minutes/day (low), 23-42 minutes/day (medium), and 42-103 minutes/day (high); diet quality using DQS: 32.5-50.0 (low), 50.0-57.5 (medium), and 57.5-72.5 (high). Sleep (hrs/day), physical activity (moderate to vigorous intensity – MVPA- minutes/day), and nutrition (Dietary Quality Score, DQS) were included in the model as a joint term. Dashed blue lines separate tertiles MVPA and dashed black lines separate tertiles of sleep. Sleep (Slp); Low Diet Quality (LD); Medium Diet Quality (MD); High Diet Quality (HD).

**
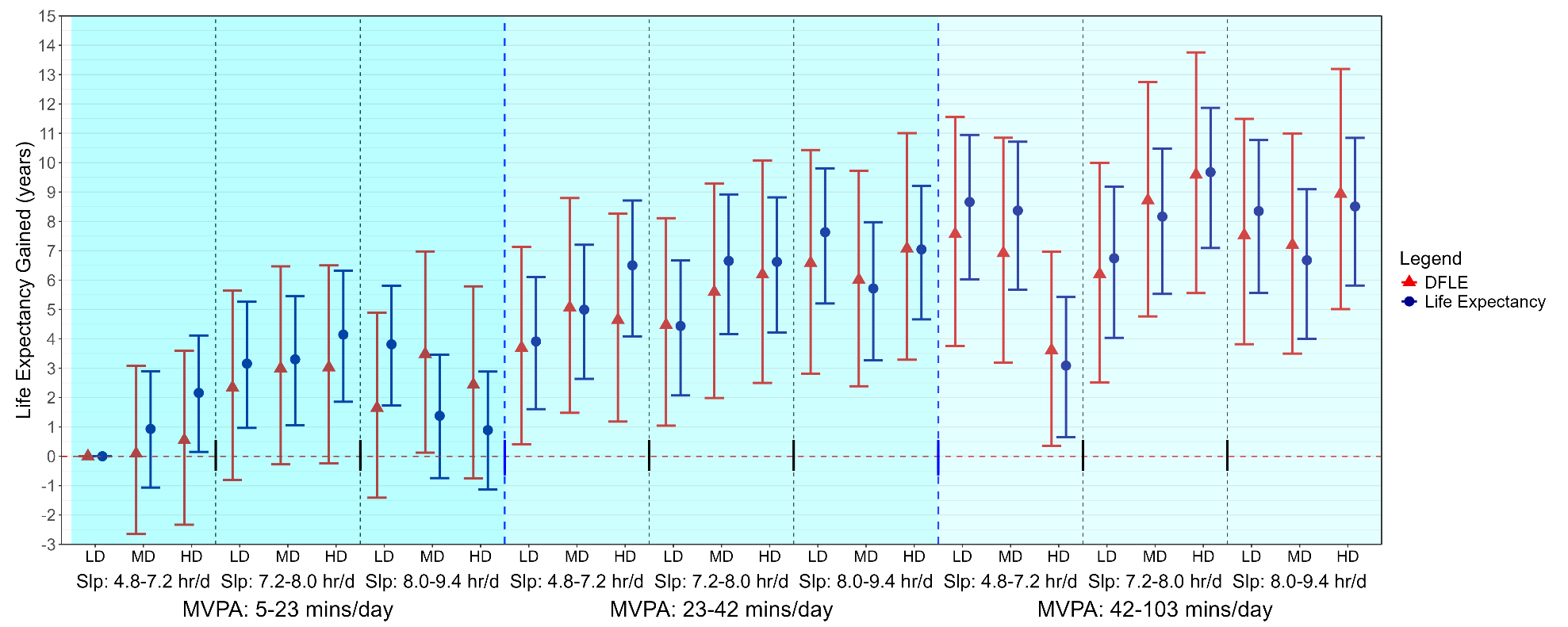
**

**Supplementary Figure 17:** Multivariable-adjusted lifespan and healthspan associated with joint sleep, physical activity, and nutrition exposures adjusted for season of accelerometery data collection (n = 59,078; all-cause mortality events = 2,458)

**Legend:** Life expectancy (lifespan) was estimated using life table models, with predictions derived from hazard ratio-adjusted mortality rates derived from the association between each mutually exclusive sleep, physical activity, and nutrition (SPAN) category and all-cause mortality. Disease-free life expectancy (DFLE; healthspan) was calculated as the expected lifespan free from cardiovascular disease, cancer, type II diabetes, chronic obstructive pulmonary disease, or dementia. DFLE incorporated a life table approach that included age-specific incidence rates for each condition. The all-cause mortality model is adjusted for age, sex, ethnicity, smoking, education, Townsend deprivation index, alcohol, discretionary screen time (time spent watching TV or using the computer outside of work), light intensity physical activity, medication (blood pressure, insulin, and cholesterol), previous diagnosis of major cardiovascular disease (defined as disease of the circulatory system, arteries, and lymph, excluding hypertension), previous diagnosis of cancer, familial history of cardiovascular disease and cancer, and season of accelerometery data collection. The specific ranges for each exposure included sleep duration as 4.8-7.2 hours/day (low), 7.2-8.0 hours/day (medium), and 8.0-9.4 hours/day (high); MVPA time: 5-23 minutes/day (low), 23-42 minutes/day (medium), and 42-103 minutes/day (high); diet quality using DQS: 32.5-50.0 (low), 50.0-57.5 (medium), and 57.5-72.5 (high). Sleep (hours/day), physical activity (moderate to vigorous intensity – MVPA- minutes/day), and nutrition (Dietary Quality Score, DQS) were included in the model as a joint term. Dashed blue lines separate tertiles MVPA and dashed black lines separate tertiles of sleep. Sleep (Slp); Low Diet Quality (LD); Medium Diet Quality (MD); High Diet Quality (HD).

**Supplementary** **Methods 1:** Additional study design details

**Study Sample and Design**

This study analyzed the data from the UK Biobank which is an ongoing prospective cohort study comprised of adults aged 40-69 at baseline (2006-2010)^5^. Participants provided informed consent and ethical approval was provided by the UK’s National Health Service, National Research Ethics Service (Ref80 11/NW/0382). Deaths were ascertained through linkage with the National Health Service Digital of England and Wales or the National Health Service Central Register and National Records of Scotland. Censoring for England, Wales, and Scotland was up to November 30th, 2022.

Hospital inpatient data was ascertained through linkage with the National Health Service Digital for England, the Information and Statistics Division for Scotland, and Secure Anonymized Information Linkage for Wales. Censoring for England and Scotland was up to October 31st, 2022 and August 31st, 2022, respectively. Censoring for Wales was up to May 31st, 2022. Cancer data linkage was obtained through national cancer registries. For England and Wales, cancer diagnosis data were followed up through 31 December 2020 and 31 December 2016 respectively, and were provided by NHS England^6^. For Scotland, cancer diagnosis data were followed up through 30 November 2021 and provided by the National Records of Scotland^6^.

Between 2013 and 2015 (median 5.5 years after the baseline measurements), 103,684 UK Biobank participants wore a wrist-worn accelerometer for 7 days^7^. The accelerometers were calibrated before being mailed to the individuals. During the initial processing stage, we excluded participants who if no sleep data was recorded, the accelerometer was poorly calibrated (>10 milli-gravitational units (mg)), or a faulty accelerometer was distributed (>100mg)^8-11^. We excluded participants with missing covariates and insufficient valid wear days. Monitoring days were considered valid if wear time was greater than 16 hours. To be included in the analysis, participants were required to have at least three valid monitoring days, with at least one of those days being a weekend day. We excluded participants who reported that they could not walk^8-11^.

**Outcome ascertainment**

Ascertainment of major chronic conditions

| **Variable** | **Definition** |
| --- | --- |
| Cardiovascular disease incidence^11^ | CVD was defined as diseases of the circulatory system, excluding hypertension, diseases of arteries, and lymph. The ICD-10 codes included were: I00, I11, I13, I20-I51, I60-I69. |
| Cancer incidence definition^12-14^ | The definition of total cancer included all cancer types. The ICD-10 codes used were C00-C97. |
| Type II diabetes^15^ | Inpatient hospitalization record ICD 10 codes: E11.  Read Codes using general practitioner records:  Version *2*: C1041, C1096, C109A, C109B, C109C, C109E, C109F, C109G, C109H, C10F6, C10FA, C10FB, C10FC, C10FE, C10FF, C10FG, C10FH, C10FL, C10FM, C10FQ, C10FR.  Version *3*: C1011, C102, C1021, C1031, C1041, C1051, C1061, C1071, C1074, C1090, C1091, C1092, C1093, C1094, C1095, C1096, C1097, C10y1, C10z1, X40J5, X40J6, X40JJ, Xaagf, XaCJ2, XaELQ, XaEnp, XaEnq, XaF05, XaFmA, XaFn7, XaFn8, XaFn9, XaFWI, XaIrf, XaIzQ, XaIzR, XaJQp, XaKyX, XE10F, XSETH. |
| Chronic Obstructive Pulmonary Disease^16^ | Inpatient hospitalization record ICD 10 codes:  J41, J43, J44, J98.2, J98.3 |
| Dementia^17^ | Inpatient hospitalization record ICD 10 codes:  A81.0, F00, F00.0, F00.1, F00.2, F00.9, F01, F01.0, F01.1, F01.2, F01.3, F01.8, F01.9, F02, F02.0, F02.1, F02.2, F02.3, F02.4, F02.8, F03, F05.1, F10.6, G30, G30.0, G30.1, G30.8, G30.9, G31.0, G31.1, G31.8, I67.3  Read Codes using general practitioner records:  Version *2*: 1461, A411, A4110, E00, E000, E001, E0010, E0011, E0012, E0013, E001z, E002, E0020, E0021, E002z, E003, E004, E0040, E0041, E0042, E0043, E004z, E012, E02y1, E041, Eu00, Eu000, Eu001, Eu002, Eu00z, Eu01  Version *3*: 1461, 1461, E11, E111, E112, E113, E114, E115, E116, E11Z, F21Z, F371, G78, A411, A4110, E00, E000, E001, E0010, E0011, E0012, E0013, E001z, E002, E0020, E0021, E002z, E003, E004, E0040, E0041 |

**Supplementary Methods 2**. Wearable behaviour classification methods

**Sleep and non-wear time**

The non-wear time was determined using a previously validated algorithm that uses wrist tilt angle to determine non-wear with 86-95% accuracy^18^. No values were imputed for non-wear time. Sleep was defined as the average daily duration of sleep (hours/day) as calculated using a validated algorithm based on relative changes in wrist tilt angle between successive 5-second windows^19^. For each interval of 5 seconds, the average of the estimated wrist tilt angle was calculated and a rolling 5-minute median served as an input for the algorithm to identify sleep onset and sleep offset, and then time spent asleep within this timeframe^18,19^.

**Two-stage random forest physical activity intensity and posture classification**

Physical activity was classified using a validated two-stage random forest activity classifier that first classifies each 10 second window (epoch) as sedentary (lying or sitting still), stationary plus (active sitting, standing still, active standing), walking, or running (**Diagram A**)^18-20^. These activities were then classified into one of four activities including: sedentary, light, moderate, and vigorous. Walking activities (gardening, active commuting, etc) were classified by normalized gravitational units (g) where <100 milli g were classified as light intensity (<3 METs), ≥100 milli g and <400 milli g were considered moderate intensity physical activity (≥3 to <6 METs), and ≥400 milli g were considered vigorous-intensity PA (≥6 METs)^20^. All windows classified as running/high energetic activity were classified as vigorous-intensity physical activity (≥ 6 METs)^8,10,20^. A major advantage of this classification approach is the lower risk of possible misclassification of sporadic high-accelerations that may occur during certain stationary light activities (e.g., dishwashing)^21-23^.

**Physical Activity Classification Scheme (Diagram A)**


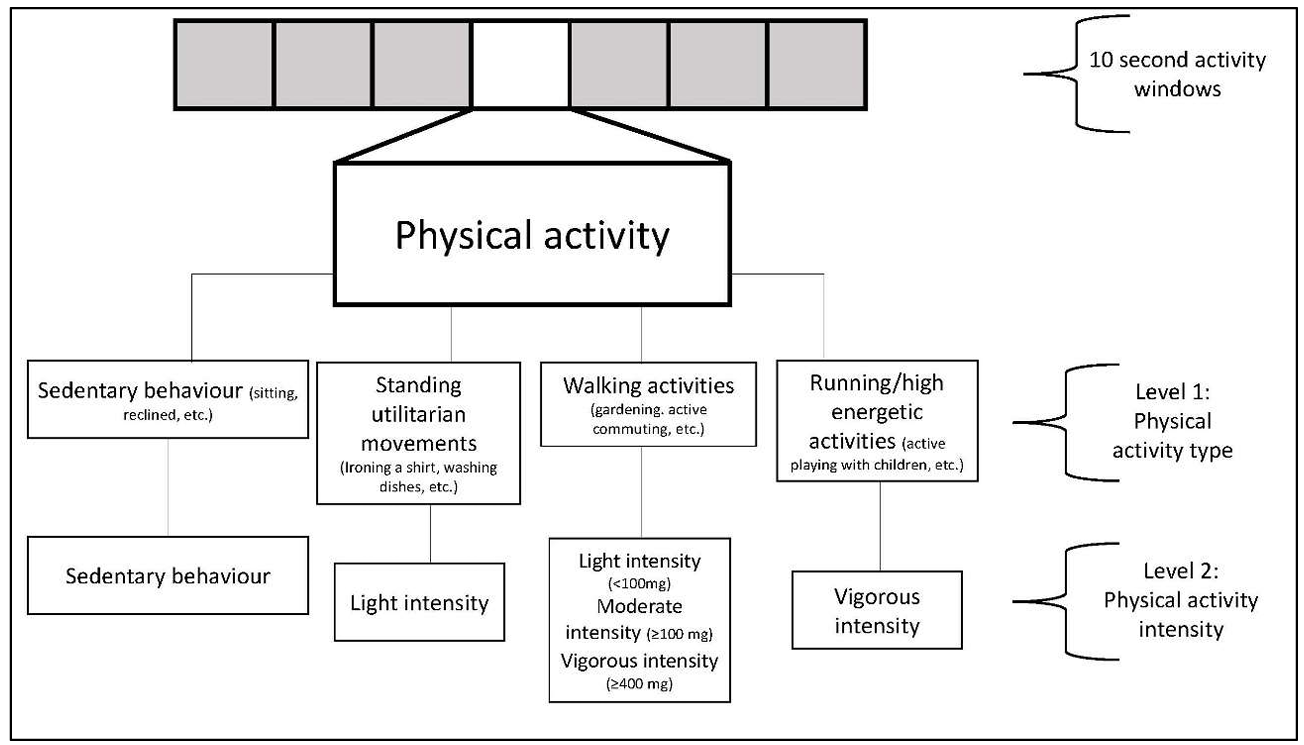


**Physical Activity Classification Performance**

The performance of this physical activity classification scheme was tested in an independent sample of 102 adults from the US^24^ and Australia^25^. This data includes direct observation measurement of 105,767 activity samples from structured and free-living activities (17,627 minutes), which were used to test the robustness and generalizability of the two-stage activity and intensity classifier. The data was collected from participant-worn or researcher-held Go-Pro video recordings. All data was imported into Noldus Observer XT software for continuous video coding. The direct observation coding generated continuous physical activity codes corresponding to the start and finish of each movement. These coded movements were then compared against the accelerometer data using the available time-stamp information. The table below includes the performance metrics across activities. Interobserver reliability was assessed by dual coding. The intraclass correlation coefficient for coding activities was 0.912 (0.866-0.942). The performance in metrics and confusion matrix for activity classification is shown below.

**Classifier Performance Metrics for Intensity in US and Australian Adults**

|  | Sensitivity | Specificity | Precision | F-score | Overall Accuracy | Weighted Kappa | Overall F-score |
| --- | --- | --- | --- | --- | --- | --- | --- |
| Sedentary | 86.5 | 93.7 | 90.5 | 88.5 |  |  |  |
| Light | 71.2 | 89.4 | 55.8 | 62.6 |  |  |  |
| Moderate | 85.4 | 96.6 | 92.7 | 88.9 |  |  |  |
| Vigorous | 95.4 | 99.4 | 94.6 | 95.0 |  |  |  |
|  |  |  |  |  | **84.6** | 0.78 | 83.8 |

Rows= ground truth; columns=predictions; bold=correct classification; all activities were free-living or simulated free-living activities.

**Confusion Matrix for Activity Classification in US and Australian Adults**

|  | Sedentary | Light | Moderate | Vigorous |
| --- | --- | --- | --- | --- |
| Sedentary | **36,904** | 5,232 | 508 | 2 |
| Light | 3,120 | **11,712** | 1,612 | 17 |
| Moderate | 502 | 4,016 | **29,528** | 526 |
| Vigorous | 226 | 17 | 214 | **9,470** |

Rows= ground truth; columns=predictions; bold=correct classification; all activities were free-living or simulated free-living activities.

**Supplementary Methods 3**. Calculation of lifespan and healthspan

Life expectancy (lifespan) and disease-free life expectancy (healthspan) were calculated using a traditional life table approach^26-29^ which use three pieces of information including:

1. All-cause mortality risk for lifestyle combinations derived from multivariable adjusted all-cause mortality model using a sample of adults from the UK Biobank wearables sub-study^30^
2. Age and sex-specific mortality rates estimated from UK Census Data^26^
3. Age-specific prevalence and incidence rates of the five leading contributors of chronic disease including cardiovascular disease, cancer, type II diabetes, chronic obstructive pulmonary disease, and dementia

Life tables can be conducting the below functions at each age in the table:

$l_{x}$: The number of people alive at the start of age $x$

$q_{x}$: The probability of dying between ages 𝑥 and 𝑥 +1

$d_{x}$: The number of deaths between ages 𝑥 and 𝑥 + 1, calculated as

$$d_{x}= l_{x}* q_{x}$$

$L_{x}$: The person-years lived between ages 𝑥 and 𝑥 +1 approximated as

$$L_{x}= l_{x}- \frac{d_{x}}{2}$$

$T_{x}$: The total person-years lived above age 𝑥, calculated as

$$T_{x}= \sum_{x} L_{x}$$

$e_{x}$: Life expectancy at age $x$, calculated:

$$e_{x}= \frac{T_{x}}{l_{x}}$$

In this study, life tables were constructed for the total sample and sex-specific groups, incorporating female- and male-specific mortality rates proportionally in the dataset and sex-stratified analyses. Mortality rates were extracted from UK Census data^26^ across sex-specific 5-year age bands. In the present study, life expectancy at age 40 and ending at age 100 was used, aligning with the age of the core analytical sample. As previously described^31^, we then fitted a multi-variable all-cause mortality model for the joint tertile-based sleep, physical activity, and nutrition (SPAN) exposure to extract hazard ratios (HR) for each mutually exclusive category. Changes in life expectancy associated with varying levels of sleep, physical activity, and nutrition were calculated by incorporating the derived HRs into the life table to reflect the change in mortality risk^27,32^. To estimate the gain in life expectancy, the predicted life expectancy for the referent group was then subtracted from the life expectancy of each SPAN combination. The same methodology was used to estimate life expectancy with the individual behaviours and the continuous SPAN score which combined these behaviours with equal weight on a scale from 0 to 100.

Disease-free life expectancy was calculated as an extension of the life table approach^26,27^, accounting for the age-specific prevalence and incidence of the five leading contributors to disease burden in the UK^33^, including cardiovascular disease, type II diabetes, cancer, and chronic obstructive pulmonary disease. The incidence in each stratified SPAN group was estimated to assess the burden of disease at each age, assuming static rates with no dynamic changes over time.

**Disease-Free Life Expectancy (healthspan): Continuation of the Life Table**

Partition years lived ($L_{x}$) into $L_{x}^{Healthy}$and $L_{x}^{Diseased}$by considering the prevalence and incidence of disease $P_{x}$ at each age ($x$):

Calculate healthy person-years at age $x$:

$$L_{x}^{Healthy}= L_{x}*{(1-P}_{x})$$

Calculate the number of diseased years at age $x$

$$L_{x}^{Diseased}= L_{x}*P_{x}$$

Sum the years of healthy living:

$T_{x}^{Healthy}=$ $\sum_{x} L_{x}^{Healthy}$

Calculate disease-free life expectancy

$e_{x}^{Healthy}=$ $\frac{T_{x}^{Healthy}}{l_{x}}$

The calculation was then performed within the joint-tertile-based SPAN analyses, where individual life tables were created for each mutually exclusive category, incorporating shifts in disease prevalence. The disease-free life expectancy was then calculated as an extension of the primary life table, partitioning the years lived at each age into those with and without chronic conditions to estimate the total years lived free of disease. To address variability and uncertainty in disease incidence and prevalence across groups, we used a parametric sampling-based Monte Carlo simulation with 10,000 iterations to estimate disease-free life expectancy^34-36^. To estimate the minimum change for disease-free life expectancy, the SPAN score was transformed back into combined SPAN components using a previously established relative contribution from each behaviour in their combined association with mortality^31^.

**Supplementary Methods 4**. Calculation of the composite SPAN score

To explore the minimum effective behaviour changes^31^ needed for meaningful improvements in lifespan and healthspan, we constructed a continuous composite SPAN score scored equally (i.e., 33.3 points per behaviour) according to the dose-response relationship of each behaviour with mortality. The scoring of each exposure within the composite SPAN score was determined based on the theoretically optimal levels identified from the dose-response relationship with all-cause mortality^37,38^.

For example, we identified a U-shaped dose-response relationship between sleep and all-cause mortality, with the lowest risk of mortality observed at 7.5 hours per day of sleep. To account for this in the score, the theoretically most optimal sleep duration of 7.5 hours would be scored 33.3 points, while sleep less or more than this amount would be scored proportionately as it deviates from this point. For physical activity and diet, the relationship was positively linear with all-cause mortality and was scored proportionally from 0-33.3 based on their relative rank according to the minimum and maximum of each behaviour. The final composite SPAN score was the sum of these three behaviour scores, ranging from 0-100, where higher values indicate theoretically healthier. The overall contribution of each behaviour was then interpreted using the hazard ratio estimates from the joint tertile-based SPAN model with all-cause mortality. The model estimates were used to derive behaviour-specific weights, which were then applied to transform the SPAN score into the incremental variations needed for improvements in lifespan and health span. This approach was undertaken to ensure the relative contribution each of each behaviour was only taken in account once, avoiding artificial amplification of behavioural importance.

**The formula is described as:**

SPAN_score =

((Physical_Activity − min(Physical_Activity)) / (max(Physical_Activity) − min(Physical_Activity))) × 33.33

+ ((Diet − min(Diet)) / (max(Diet) − min(Diet))) × 33.33

+ [33.33 − (|Sleep − 7.5| / max(|Sleep − 7.5|)) × 33.33]

**Supplementary Table 1.** Diet quality score index for food-frequency questionnaire dietary data

| **Food components** | **UK Biobank field ID** | **Amount per serving** | **Criteria for maximum score (10)** | **Criteria for minimum score (0)** |
| --- | --- | --- | --- | --- |
| Fruit | 1309 (pieces fresh fruit/day) 1319 (pieces dried fruit/day) | 1309 – 1 piece  1319 – 5 pieces | ≥3 servings/day | 0 servings/day |
| Vegetable | 1289 (tablespoons cooked vegetables/day)  1299 (salad/raw vegetables/day) | 3 heaped tablespoons | ≥3 servings/day | 0 servings/day |
| Whole grains | 1438, 1448 (wholemeal/wholegrain bread slices/week)  1458, 1468 (bran/oat/muesli cereal) | 1438/1448 – 1 slice/day 1458/1468 – 1 bowl/day | ≥3 servings/day | 0 servings/day |
| Fish | 1329 (oily fish/week)  1339 (non-oily fish/week) | Once/week | ≥2 servings/week | 0 servings/week |
| Dairy | 1408 (cheese/week)  1418 (milk type) | 1408 – 1 piece/day  1418 – 1 glass/day if consumption of any type of milk | ≥2 servings/day | 0 servings/day |
| Vegetable oils | 1428 (Flora Pro-Active/Benecol spread)  2654 (Flora Pro-Active/Benecol, soft margarine -, olive oil based -, polyunsaturated/sunflower oil based -, other low/reduced fat spread)  1438 (bread slices/week) | 1 serving/day if in combination with eating at least 2 slices of bread (ID 1438) | ≥2 servings/day | 0 servings/day |
| Refined grains | 1438, 1448 (white, brown, other bread slices/week) 1458, 1468 (biscuit, other cereals/week) | 1438/1448 – 1 slice/day 1458/1468 – 1 bowl/day | 0 servings/day | >2 servings/day |
| Processed meats | 1349 (processed meat/week or daily)  3680 (age when last ate meat) | 1349 – 1 piece/day  3680 – 0 pieces/day if indicated having never eaten meat | 0 serving/week | >1 serving/week |
| Unprocessed red meats | 1369 (beef/week or day)  1379 (lamb or mutton/week or day)  1389 (pork/week or day)  3680 (age when last ate meat) | 1359-1389 – once/week 3680 – 0 pieces/day if indicated having never eaten meat | 0 serving/week | >2 serving/week |
| Sugar-sweetened beverages | 6144 (never consumes drinks containing sugar) | 0 servings | Don’t drink | Drink |

Diet quality score information is adapted from previously established work by Zhuang *et al*. Diabetes Care^39^. Intermediate intake for each dietary component were scored relative to minimum to maximum intake of each food component. The formula for intermediate intakes of adequacy components is described as: component score = (maximum score / (Amax - Amin))*(X - Amin) and for moderate components (refined grains, processed meat, and unprocessed red meat) component score = (maximum score - maximum score / (Amax - Amin))*(X - Amin). The food frequency questionnaire demonstrated moderate reproducibility for food groups (Intraclass Correlation Coefficient (ICC): 0.48-0.66) and modest agreement with alternative dietary intake measures from the 24-hour recall (ICC: 0.38-0.63). This level of agreement and reproducibility is comparable to previous prospective observational studies^40-42^. The food frequency questionnaire has also been validated against the 24-hour dietary recall using objective biomarkers as the standard^43^.

**Supplementary Table 2:** Mortality and disease events across the mutually exclusive sleep, physical activity, and nutrition combinations

|  |  | **Nutrition Low** | **Nutrition Medium** | **Nutrition High** |
| --- | --- | --- | --- | --- |
| **MVPA Low** | **Sleep Low** | n = 2542 (Events = 203) | n = 2234 (Events = 163) | n = 2205 (Events = 166) |
|  | **Sleep Medium** | n = 2178 (Events = 116) | n = 1982 (Events = 100) | n = 1861 (Events = 100) |
|  | **Sleep High** | n = 2458 (Events = 138) | n = 2136 (Events = 138) | n = 2097 (Events = 152) |
| **MVPA Medium** | **Sleep Low** | n = 2312 (Events = 98) | n = 2116 (Events = 83) | n = 1945 (Events = 77) |
|  | **Sleep Medium** | n = 2298 (Events = 85) | n = 2241 (Events = 75) | n = 2092 (Events = 69) |
|  | **Sleep High** | n = 2339 (Events = 67) | n = 2180 (Events = 66) | n = 2169 (Events = 73) |
| **MVPA High** | **Sleep Low** | n = 2271 (Events = 48) | n = 2062 (Events = 50) | n = 2006 (Events = 92) |
|  | **Sleep Medium** | n = 2407 (Events = 55) | n = 2386 (Events = 52) | n = 2247 (Events = 46) |
|  | **Sleep High** | n = 2113 (Events = 46) | n = 2127 (Events = 56) | n = 2074 (Events = 44) |

The sample size and number of all-cause mortality events for each Sleep, Physical Activity, and Nutrition group is detailed above (n = 59,078; events = 2,458). Participants were grouped by Sleep, Physical Activity, and Nutrition exposure tertiles (i.e., low, moderate, and high) which equated to a joint exposure of 27 separate groups for all three behaviours. The specific ranges for each exposure included sleep duration as 4.8-7.2 hours/day (low), 7.2-8.0 hours/day (medium), and 8.0-9.4 hours/day (high); moderate to vigorous physical activity (MVPA) measurements as 5-23 minutes/day (low), 23-42 minutes/day (medium), and 42-103 minutes/day (high); and diet quality using the DQS as 32.5-50.0 (low), 50.0-57.5 (medium), and 57.5-72.5 (high).

**Supplementary Table 3.** Covariate Definitions.

| **Variable** | **Definition** | **UK Biobank field ID (if applicable)** |
| --- | --- | --- |
| Age | Categorical (4) | 34, 52, accelerometer date-timestamp |
| Sex | Female/Male | 31 |
| Ethnicity | White/Others | 21000 |
| Education | College/University; A/AS level; O levels; CSE; NVQ/HND/HNC; other | 6138 |
| Smoking status | Never, past, current | 20116 |
| Alcohol consumption | Units/day | 20403 |
| Light intensity physical activity | Standing utilitarian movements, slow walking (<3 METs) | Derived from accelerometer data |
| Discretionary screen-time | Self-reported time spent/day watching TV and using a computer outside of work | 1070, 1080 |
| Townsend deprivation | Categorical (5) | 22189 |
| Use of cholesterol medication | Yes/No | 6177, 6153 |
| Use of blood pressure medication | Yes/No | 6177, 6153 |
| Use of diabetes medication | Yes/No | 6177, 6153 |
| Previous CVD | Identified by self-report and hospitalization. Defined as disease of the circulatory system, arteries, and lymph, excluding hypertension | 20002, 41270 |
| Previous cancer | Identified by self-report and cancer registry. | 20001, 100092 |
| Familial history of CVD | Self-reporter mother of father diagnosed with heart disease or stroke | 20107, 20110 |
| Familial history of cancer | Self-reporter mother of father diagnosed with cancer | 20107, 20110 |
| High frailty scale | Categorical (yes/no); high frailty indicates a score of ≥3 on a 0 to 5 | 2306, 120107, 2624, 1011, 3637, 991, 971, 924, 46, 47 |
| Body mass index | Continuous; kilogram/meter^2^ | 23104 |
| Total energy intake | Continuous, kcal/day derived from 24-hour dietary recall data | 26002 |
| Morning/evening person (chronotype) | Categorical (definitely a ‘morning’ person; more a ‘morning’ person than ‘evening’ person; more an ‘evening’ person than ‘morning’ person; definitely an ‘evening’ person) | 1180 |
| Insomnia | Categorical (never/rarely; sometimes; usually) | 1200 |
| Snoring | Categorical (yes/no) | 1210 |
| Daytime sleepiness | Categorical (never/rarely; sometimes; often) | 1220 |

Additional detail is available online at <https://biobank.ndph.ox.ac.uk/showcase/>.

**Supplementary Table 4:** Model variance inflation factors for combined SPAN behaviours

| **Primary Model with Combined SPAN Behaviours** | |
| --- | --- |
| **Variable** | **Variance inflation factor (VIF)** |
| Sleep, moderate to vigorous physical activity, and nutrition (combined variable) | 1.28 |
| Age (self-report) | 1.17 |
| Sex (self-report) | 1.17 |
| Ethnicity (self-report) | 1.03 |
| Smoking (self-report) | 1.10 |
| Alcohol (self-report) | 1.15 |
| Education (self-report) | 1.08 |
| Socioeconomic status (self-report) | 1.06 |
| Light physical activity (accelerometry derived) | 1.14 |
| Previous CVD (self-report) | 1.15 |
| Previous cancer (self-report) | 1.03 |
| Familial history of CVD (self-report) | 1.01 |
| Familial history of cancer (self-report) | 1.01 |
| Discretionary screen time (self-report) | 1.09 |
| Medication (self-report) | 1.21 |

The table provides the variance inflation factor (VIF) for each covariate in the primary analytical model (combined SPAN behaviours) adjusted for self-reported discretionary screen time and the sensitivity model adjusted for accelerometery derived sedentary behaviour. VIF values measure multicollinearity among the exposure variables, with a value of 1 indicating no correlation with other predictors. Higher values suggest increasing multicollinearity, with values greater than 5 indicating potentially problematic multicollinearity^44^.

**Supplementary Table 5.** NOVA classification of food groups for 24-hour dietary recall data.

| **NOVA classification level** | **UK Biobank field ID (if applicable)** |
| --- | --- |
| Level four | Added sugars and preserves (26064), Animal fat spread lower fat (26062), Animal fat spread normal (26063), Biscuit cereal (26075), Biscuits (26068), Bran cereal (26076), Breaded/battered chicken (26069), Breaded/battered fish (26070), Chocolate confectionery (26080), Cream (26154), Fried/roast potatoes (26119), Low/non sugar sugar-sweetened beverages (26126), Mashed potatoes (26120), Meat substitutes - soy (26137), Meat substitutes - vegetarian (26145), Milk-based and powdered drinks (26087), Milk-dairy desserts (26084), Mixed bread brown and seeded (26071), Muesli (26105), Nut-based spreads (26106), Other cereal (sugar) (26079), Other desserts and cakes and pastries (26085), Other sweets (26140), Pizza (26116), Plant-based spread lower fat (26111), Plant-based spread normal (26112), Processed meat (26122), Samosa, pakora (26128), Sauces and condiments (high fat) (26129), Sauces and condiments (low fat) (26130), Savory crackers (26083), Savory snacks (26134), Soy desserts and yogurt (26086), Sugar-sweetened beverages and other sugary drinks (26127), Sushi (26139), Vegetable dips (26144) |
| Level three | High fat cheese (26099), Medium and low fat cheese (26103), White fish and tinned tuna (26149), White bread (26073), Wholemeal bread (26074), Other bread (26072) |
| Level two | Grain dishes - added fat (26097), Olive oil (drizzling/dunking) (26110) |
| Level one | Allium vegetables (26065), Apples and pears (26089), Beef (26066), Berries (26090), Citrus (26091), Coffee, caffeinated (26081), Coffee, decaffeinated (26082), Dried fruit (26092), Egg and egg dishes (26088), Fruit juice (26095), Green leafy/cabbages (26098), Lamb (26100), Legumes and pulses (26101), Oat cereal (non sugar) (26077), Oat cereal (sugar) (26078), Low fat yogurt (26102), Full fat yogurt (26096), Oily fish (26109), Other fruit (26093), Other meat, offal (26104), Other vegetables, including mushrooms, fruiting and mixed vegetables (26146), Peas and sweetcorn (26115), Pork (26117), Potatoes and sweet potatoes (baked/boiled) (26118), Poultry (26121), Raw salad (26123), Root vegetables (26125), Salted nuts and seeds (26108), Semi skimmed milk (26131), Rice/oat milk (26124), Shellfish (26132), Skimmed milk and cholesterol-lowering milk (26133), Soups (26135), Soy milk (26136), Stewed fruit (26094), Tea (26141), Tea, decaffeinated (26142), Tomatoes (26143), Unsalted nuts and seeds (26107), White pasta and rice (26113), Whole milk (26150), Wholemeal pasta, brown rice and other wholegrains (26114) |

From 2009-2012, dietary data was also collected using 1-4 separate 24-hour dietary recall for a subgroup of participants (n = 211,031)^2^. Additional detail on reproducibility and agreement between FFQ and the 24-hour dietary recall has been published elsewhere^45,46^. Food groups in each NOVA classification level were reported as the average weight (gram/day) from the 24-hour dietary recalls. Ultra-processed food intake was defined as the percentage of level four NOVA food groups relative to the average reported total food weight. All food categories and the definition of ultra-processed food intake were determined using a previously established method^47,48^.

**Supplementary Table 6:** Participant characteristics

|  | **Overall** | **Sleep** | | | **Physical activity** | | | **Nutrition** | | | |
| --- | --- | --- | --- | --- | --- | --- | --- | --- | --- | --- | --- |
|  |  | **Low** | **Moderate** | **High** | **Low** | **Moderate** | **High** | **Low** | **Moderate** | **High** |  |
| **Sample** | 59,078 | 19,693 | 19,692 | 19,693 | 19,693 | 19,692 | 19,693 | 20,918 | 19,464 | 18,696 |  |
| **All-cause mortality events, n** | 2,458 | 980 | 698 | 780 | 1,276 | 693 | 489 | 856 | 783 | 819 |  |
| **Follow up, years** | 8.06 [7.49, 8.59] | 8.05 [7.48, 8.56] | 8.07 [7.51, 8.59] | 8.05 [7.51, 8.59] | 8.05 [7.46, 8.59] | 8.06 [7.52, 8.59] | 8.07 [7.52, 8.56] | 8.06 [7.50, 8.59] | 8.06 [7.50, 8.59] | 8.05 [7.49, 8.56] |  |
| **Cardiovascular disease events, n** | 9,996 | 3,792 | 3,090 | 3,114 | 4,395 | 3,158 | 2,443 | 3,381 | 3,227 | 3,388 |  |
| **Cancer events, n** | 7,681 | 2,563 | 2,425 | 2,693 | 3,043 | 2,499 | 2,139 | 2,665 | 2,537 | 2,479 |  |
| **Type II Diabetes events, n** | 2,971 | 1,346 (6.8%) | 814 (4.1%) | 811 (4.1%) | 1,654 (8.4%) | 814 (4.1%) | 503 (2.6%) | 1,087 (5.2%) | 827 (4.2%) | 1,057 (5.7%) |  |
| **COPD events, n** | 1,540 | 634 (3.2%) | 466 (2.4%) | 440 (2.2%) | 833 (4.2%) | 424 (2.2%) | 283 (1.4%) | 635 (3.0%) | 453 (2.3%) | 452 (2.4%) |  |
| **Dementia events, n** | 508 | 225 (1.1%) | 130 (0.7%) | 153 (0.8%) | 270 (1.4%) | 147 (0.7%) | 91 (0.5%) | 155 (0.7%) | 158 (0.8%) | 195 (1.0%) |  |
| **Age, years** | 64.00 [57.00, 69.00] | 64.00 [57.00, 69.00] | 63.00 [56.00, 68.00] | 64.00 [57.00, 69.00] | 66.00 [60.00, 70.00] | 64.00 [57.00, 69.00] | 62.00 [55.00, 67.00] | 62.00 [55.00, 68.00] | 64.00 [57.00, 69.00] | 65.00 [59.00, 70.00] |  |
| **Male, %** | 26,810 (45.4%) | 10,068 (51.1%) | 10,822 (55.0%) | 11,378 (57.8%) | 8,445 (42.9%) | 9,002 (45.7%) | 9,363 (47.5%) | 10,582 (50.6%) | 8,115 (41.7%) | 8,113 (43.4%) |  |
| **Sleep, hours, (median [IQR])** | 7.62 [6.89, 8.26] | 6.52 [5.92, 6.89] | 7.62 [7.41, 7.83] | 8.52 [8.26, 8.89] | 7.60 [6.78, 8.30] | 7.65 [6.92, 8.27] | 7.62 [6.94, 8.22] | 7.61 [6.86, 8.26] | 7.62 [6.90, 8.25] | 7.64 [6.90, 8.27] |  |
| **Moderate to vigorous physical activity, (median [IQR])** | 31.26 [18.52, 49.09] | 30.17 [17.52, 48.19] | 33.05 [19.79, 51.31] | 30.60 [18.24, 47.79] | 14.31 [9.64, 18.52] | 31.26 [26.76, 36.26] | 58.74 [49.09, 74.91] | 30.57 [18.24, 48.31] | 31.67 [18.76, 49.43] | 31.60 [18.59, 49.64] |  |
| **Diet quality score, (median [IQR])** | 54.29 [47.50, 60.00] | 53.93 [47.50, 60.00] | 54.01 [47.50, 60.00] | 54.64 [47.50, 60.00] | 53.93 [47.50, 60.00] | 54.29 [47.50, 60.00] | 54.29 [47.50, 60.00] | 45.00 [40.00, 47.50] | 55.00 [52.50, 56.79] | 62.50 [60.00, 67.50] |  |
| **Light physical activity, (median [IQR])** | 102.84 [70.24, 158.12] | 106.72 [72.26, 162.46] | 105.17 [72.00, 162.25] | 96.81 [66.72, 149.53] | 83.96 [59.79, 118.6] | 107.43 [73.38, 157.72] | 130.86 [81.72, 195.7] | 100.81 [69.03, 155.55] | 103.91 [71.26, 158.90] | 103.98 [70.53, 159.82] |  |
| **Discretionary screen time, hours, (median [IQR])** | 3.50 [2.50, 5.00] | 4.00 [2.50, 5.00] | 3.50 [2.50, 5.00] | 3.50 [2.50, 5.00] | 4.00 [3.00, 5.00] | 3.50 [2.50, 5.00] | 3.00 [2.00, 4.50] | 4.00 [2.50, 5.00] | 3.50 [2.50, 5.00] | 3.50 [2.50, 5.00] |  |
| **Smoking history, %** | ─ | ─ | ─ | ─ | ─ | ─ | ─ | ─ | ─ | ─ |  |
| **Never** | 34,024 (57.6%) | 10,910 (55.4%) | 11,508 (58.4%) | 11,606 (58.9%) | 10,781 (54.7%) | 11,462 (58.2%) | 11,781 (59.8%) | 11,928 (57.0%) | 11,385 (58.5%) | 10,711 (57.3%) |  |
| **Former** | 21,361 (36.2%) | 7,320 (37.2%) | 7,026 (35.7%) | 7,015 (35.6%) | 7,390 (37.5%) | 7,076 (35.9%) | 6,895 (35.0%) | 7,236 (34.6%) | 7,039 (36.2%) | 7,086 (37.9%) |  |
| **Current** | 3,693 (6.3%) | 1,463 (7.4%) | 1,158 (5.9%) | 1,072 (5.4%) | 1,522 (7.7%) | 1,154 (5.9%) | 1,017 (5.2%) | 1,754 (8.4%) | 1,040 (5.3%) | 899 (4.8%) |  |
| **Alcohol consumption, units (median [IQR])** | 9.75 [1.70, 19.50] | 9.52 [0.97, 19.50] | 9.75 [2.44, 19.50] | 9.75 [2.27, 19.57] | 8.77 [0.62, 18.84] | 9.75 [2.27, 19.50] | 10.71 [3.20, 20.52] | 9.75 [1.22, 20.13] | 9.75 [2.44, 19.50] | 9.75 [1.94, 19.50] |  |
| **Total energy intake, kcal/day (median [IQR])^a^** | 2,018 [1,651, 2,443] | 2,056 [1,676, 2,500] | 2,026 [1,662, 2,441] | 1,975 [1,617, 2,392] | 1,965 [1,607, 2,382] | 2,011 [1,649, 2,435] | 2,075 [1,699, 2,511] | 2,045 [1,677, 2,490] | 2,017 [1,652, 2,433] | 1,989 [1,620, 2,413] |  |
| **Townsend Deprivation Index, (median [IQR]])** | -2.49 [-3.84, -0.26] | -2.33 [-3.76, 0.13] | -2.50 [-3.83, -0.34] | -2.64 [-3.91, -0.59] | -2.41 [-3.78, -0.12] | -2.54 [-3.88, -0.34] | -2.52 [-3.85, -0.31] | -2.44 [-3.81, -0.09] | -2.52 [-3.87, -0.40] | -2.52 [-3.84, -0.33] |  |
| **Education, %** | ─ | ─ | ─ | ─ | ─ | ─ | ─ | ─ | ─ | ─ |  |
| **College/University** | 7,707 (13.0%) | 2,615 (13.3%) | 2,533 (12.9%) | 2,559 (13.0%) | 2,523 (12.8%) | 2,605 (13.2%) | 2,579 (13.1%) | 2,740 (13.1%) | 2,636 (13.5%) | 2,331 (12.5%) |  |
| **A/AS** | 25,619 (43.4%) | 8,642 (43.9%) | 8,882 (45.1%) | 8,095 (41.1%) | 8,095 (41.1%) | 8,725 (44.3%) | 8,799 (44.7%) | 8,574 (41.0%) | 8,780 (45.1%) | 8,265 (44.2%) |  |
| **O levels** | 3,262 (5.5%) | 1,090 (5.5%) | 1,065 (5.4%) | 1,107 (5.6%) | 1,145 (5.8%) | 1,097 (5.6%) | 1,020 (5.2%) | 1,264 (6.0%) | 989 (5.1%) | 1,009 (5.4%) |  |
| **CSE** | 12,028 (20.4%) | 3,897 (19.8%) | 3,923 (19.9%) | 4,208 (21.4%) | 4,104 (20.8%) | 3,982 (20.2%) | 3,942 (20.0%) | 4,422 (21.1%) | 3,896 (20.0%) | 3,710 (19.8%) |  |
| **NVQ/HND/HNC** | 2,216 (3.8%) | 716 (3.6%) | 714 (3.6%) | 786 (4.0%) | 642 (3.3%) | 685 (3.5%) | 889 (4.5%) | 943 (4.5%) | 669 (3.4%) | 604 (3.2%) |  |
| **Other** | 8,246 (14.0%) | 2,733 (13.9%) | 2,575 (13.1%) | 2,938 (14.9%) | 3,184 (16.2%) | 2,598 (13.2%) | 2,464 (12.5%) | 2,975 (14.2%) | 2,494 (12.8%) | 2,777 (14.9%) |  |
| **Parental history of CVD, %** | 33,120 (56.1%) | 11,074 (56.2%) | 10,976 (55.7%) | 11,070 (56.2%) | 11,510 (58.4%) | 11,004 (55.9%) | 10,606 (53.9%) | 11,144 (53.3%) | 11,050 (56.8%) | 10,926 (58.4%) |  |
| **Parental history of cancer, %** | 18,549 (31.4%) | 6,177 (31.4%) | 6,150 (31.2%) | 6,222 (31.6%) | 6,162 (31.3%) | 6,252 (31.7%) | 6,135 (31.2%) | 6,509 (31.1%) | 6,167 (31.7%) | 5,873 (31.4%) |  |
| **Previous CVD, %** | 5,678 (9.6%) | 2,132 (10.8%) | 1,754 (8.9%) | 1,792 (9.1%) | 2,668 (13.5%) | 1,732 (8.8%) | 1,278 (6.5%) | 1,971 (9.4%) | 1,796 (9.2%) | 1,911 (10.2%) |  |
| **Previous Cancer, %** | 5,145 (8.7%) | 1,676 (8.5%) | 1,672 (8.5%) | 1,797 (9.1%) | 2,036 (10.3%) | 1,715 (8.7%) | 1,394 (7.1%) | 1,709 (8.2%) | 1,737 (8.9%) | 1,699 (9.1%) |  |
| **Ethnicity, %** | ─ | ─ | ─ | ─ | ─ | ─ | ─ | ─ | ─ | ─ |  |
| **Other** | 3,565 (6.0%) | 1,552 (7.9%) | 1,115 (5.7%) | 898 (4.6%) | 1,160 (5.9%) | 1,162 (5.9%) | 1,243 (6.3%) | 1,257 (6.0%) | 1,186 (6.1%) | 1,122 (6.0%) |  |
| **White** | 55,513 (94.0%) | 18,141 (92.1%) | 18,577 (94.3%) | 18,795 (95.4%) | 18,533 (94.1%) | 18,530 (94.1%) | 18,450 (93.7%) | 19,661 (94.0%) | 18,278 (93.9%) | 17,574 (94.0%) |  |
| **Medication Use, %** | ─ | ─ | ─ | ─ | ─ | ─ | ─ | ─ | ─ | ─ |  |
| **Cholesterol** | 9,130 (15.5%) | 3,426 (17.4%) | 2,756 (14.0%) | 2,948 (15.0%) | 4,200 (21.3%) | 2,870 (14.6%) | 2,060 (10.5%) | 2,835 (13.6%) | 2,855 (14.7%) | 3,440 (18.4%) |  |
| **Blood pressure** | 5,384 (9.1%) | 1,997 (10.1%) | 1,680 (8.5%) | 1,707 (8.7%) | 2,287 (11.6%) | 1,723 (8.7%) | 1,374 (7.0%) | 1,782 (8.5%) | 1,749 (9.0%) | 1,853 (9.9%) |  |
| **Insulin** | 72 (0.1%) | 21 (0.1%) | 29 (0.1%) | 22 (0.1%) | 22 (0.1%) | 27 (0.1%) | 23 (0.1%) | 23 (0.1%) | 18 (0.1%) | 31 (0.2%) |  |
| **Frailty Index >3, %** | 196 (0.3%) | 95 (0.5%) | 51 (0.3%) | 50 (0.3%) | 142 (0.8%) | 24 (0.1%) | 30 (0.2%) | 91 (0.5%) | 53 (0.3%) | 52 (0.3%) |  |
| **Self-rated health, %** | ─ | ─ | ─ | ─ | ─ | ─ | ─ | ─ | ─ | ─ |  |
| **Excellent** | 12,827 (21.8%) | 3,930 (20.0%) | 4,534 (23.1%) | 4,363 (22.2%) | 3,148 (16.0%) | 4,382 (22.3%) | 5,297 (26.9%) | 4,138 (19.8%) | 4,322 (22.2%) | 4,367 (23.4%) |  |
| **Good** | 35,514 (60.2%) | 11,533 (58.7%) | 11,970 (60.9%) | 12,011 (61.1%) | 11,583 (58.9%) | 12,099 (61.5%) | 11,832 (60.2%) | 12,421 (59.5%) | 11,897 (61.2%) | 11,196 (60.0%) |  |
| **Fair** | 9,175 (15.6%) | 3,563 (18.1%) | 2,774 (14.1%) | 2,838 (14.4%) | 4,083 (20.8%) | 2,803 (14.3%) | 2,289 (11.6%) | 3,654 (17.5%) | 2,814 (14.5%) | 2,707 (14.5%) |  |
| **Poor** | 1,458 (2.5%) | 634 (3.2%) | 378 (1.9%) | 446 (2.3%) | 844 (4.3%) | 374 (1.9%) | 240 (1.2%) | 660 (3.2%) | 394 (2.0%) | 404 (2.2%) |  |
| **Body mass index, kg/m^2^** | 26.10 [23.60, 29.00] | 26.70 [24.10, 29.90] | 25.80 [23.50, 28.70] | 25.70 [23.30, 28.50] | 27.20 [24.50, 30.50] | 26.00 [23.60, 28.80] | 25.20 [23.00, 27.70] | 26.40 [23.90, 29.40] | 26.00 [23.60, 28.90] | 25.80 [23.40, 28.70] |  |

Values represent mean (SD) for the full sample unless stated otherwise stated. The table presents the participant characteristics of those with no missing data.

**References**

1. Andersson T, Alfredsson L, Källberg H, Zdravkovic S, Ahlbom A. Calculating measures of biological interaction. *European Journal of Epidemiology*. 2005;20(7):575-9. doi:10.1007/s10654-005-7835-x

2. Perez-Cornago A, Pollard Z, Young H, et al. Description of the updated nutrition calculation of the Oxford WebQ questionnaire and comparison with the previous version among 207,144 participants in UK Biobank. *European Journal of Nutrition*. 2021;60(7):4019-4030. doi:10.1007/s00394-021-02558-4

3. Tukey JW. Exploratory data analysis. *Reading/Addison-Wesley*. 1977;

4. Austin PC, White IR, Lee DS, van Buuren S. Missing Data in Clinical Research: A Tutorial on Multiple Imputation. *Canadian Journal of Cardiology*. 2021/09/01/ 2021;37(9):1322-1331.

5. Ramakrishnan R, Doherty A, Smith-Byrne K, et al. Accelerometer measured physical activity and the incidence of cardiovascular disease: Evidence from the UK Biobank cohort study. *PLOS Medicine*. 2021;18(1):e1003487. doi:10.1371/journal.pmed.1003487

6. Conroy MC, Lacey B, Bešević J, et al. UK Biobank: a globally important resource for cancer research. *Br J Cancer*. Feb 2023;128(4):519-527. doi:10.1038/s41416-022-02053-5

7. Doherty A, Jackson D, Hammerla N, et al. Large scale population assessment of physical activity using wrist worn accelerometers: the UK biobank study. *PloS one*. 2017;12(2):e0169649.

8. Stamatakis E, Ahmadi MN, Gill JMR, et al. Association of wearable device-measured vigorous intermittent lifestyle physical activity with mortality. *Nature Medicine*. 2022;28(12):2521-2529. doi:10.1038/s41591-022-02100-x

9. Stamatakis E, Ahmadi MN, Friedenreich CM, et al. Vigorous Intermittent Lifestyle Physical Activity and Cancer Incidence Among Nonexercising Adults: The UK Biobank Accelerometry Study. *JAMA Oncology*. 2023;9(9):1255-1259. doi:10.1001/jamaoncol.2023.1830

10. Ahmadi MN, Hamer M, Gill JM, et al. Brief bouts of device-measured intermittent lifestyle physical activity and its association with major adverse cardiovascular events and mortality in people who do not exercise: a prospective cohort study. *The Lancet Public Health*. 2023;8(10):e800-e810.

11. Ahmadi MN, Clare PJ, Katzmarzyk PT, del Pozo Cruz B, Lee IM, Stamatakis E. Vigorous physical activity, incident heart disease, and cancer: how little is enough? *European Heart Journal*. 2022;43(46):4801-4814.

12. Ahmad AS, Offman J, Delon C, North BV, Shelton J, Sasieni PD. Years of life lost due to cancer in the United Kingdom from 1988 to 2017. *British Journal of Cancer*. 2023;129(10):1558-1568. doi:10.1038/s41416-023-02422-8

13. Thomson B, Emberson J, Lacey B, Lewington S, Peto R, Islami F. Association of smoking initiation and cessation across the life course and cancer mortality: prospective study of 410 000 US adults. *JAMA oncology*. 2021;7(12):1901-1903.

14. Feng H, Yang L, Ai S, et al. Association between accelerometer-measured amplitude of rest activity rhythm and future health risk: a prospective cohort study of the UK Biobank. *The Lancet Healthy Longevity*. 2023;4(5):e200-e210. doi:10.1016/S2666-7568(23)00056-9

15. Boonpor J, Petermann-Rocha F, Parra-Soto S, et al. Types of diet, obesity, and incident type 2 diabetes: Findings from the UK Biobank prospective cohort study. *Diabetes Obes Metab*. Jul 2022;24(7):1351-1359. doi:10.1111/dom.14711

16. He Y, Qian DC, Diao JA, et al. Prediction and stratification of longitudinal risk for chronic obstructive pulmonary disease across smoking behaviors. *Nature Communications*. 2023/12/14 2023;14(1):8297. doi:10.1038/s41467-023-44047-8

17. Wilkinson T, Schnier C, Bush K, et al. Identifying dementia outcomes in UK Biobank: a validation study of primary care, hospital admissions and mortality data. *Eur J Epidemiol*. Jun 2019;34(6):557-565. doi:10.1007/s10654-019-00499-1

18. Ahmadi MN, Nathan N, Sutherland R, Wolfenden L, Trost SG. Non-wear or sleep? Evaluation of five non-wear detection algorithms for raw accelerometer data. *Journal of Sports Sciences*. 2020;38(4):399-404.

19. van Hees VT, Sabia S, Jones SE, et al. Estimating sleep parameters using an accelerometer without sleep diary. *Scientific Reports*. 2018;8(1):12975. doi:10.1038/s41598-018-31266-z

20. Pavey TG, Gilson ND, Gomersall SR, Clark B, Trost SG. Field evaluation of a random forest activity classifier for wrist-worn accelerometer data. *Journal of Science and Medicine in Sport*. 2017;20(1):75-80. doi:10.1016/j.jsams.2016.06.003

21. Trost SG, Fragala-Pinkham M, Lennon N, O'Neil ME. Decision Trees for Detection of Activity Intensity in Youth with Cerebral Palsy. *Med Sci Sports Exerc*. May 2016;48(5):958-66. doi:10.1249/mss.0000000000000842

22. Ahmadi MN, Trost SG. Device-based measurement of physical activity in pre-schoolers: Comparison of machine learning and cut point methods. *PLoS One*. 2022;17(4):e0266970.

23. CMT VANL, Okely AD, Batterham MJ, et al. Wrist Acceleration Cut Points for Moderate-to-Vigorous Physical Activity in Youth. *Med Sci Sports Exerc*. Mar 2018;50(3):609-616. doi:10.1249/mss.0000000000001449

24. Reiss A, Weber M, Stricker D. Exploring and extending the boundaries of physical activity recognition. 2011:46-50.

25. Clark BK, Winkler EA, Brakenridge CL, Trost SG, Healy GN. Using Bluetooth proximity sensing to determine where office workers spend time at work. *PLOS ONE*. 2018;13(3):e0193971. doi:10.1371/journal.pone.0193971

26. Ferrari AJ, Santomauro DF, Aali A, et al. Global incidence, prevalence, years lived with disability (YLDs), disability-adjusted life-years (DALYs), and healthy life expectancy (HALE) for 371 diseases and injuries in 204 countries and territories and 811 subnational locations, 1990–2021: a systematic analysis for the Global Burden of Disease Study 2021. *The Lancet*. 2024;403(10440):2133-2161.

27. Sun Q, Yu D, Fan J, et al. Healthy lifestyle and life expectancy at age 30 years in the Chinese population: an observational study. *The Lancet Public Health*. 2022;7(12):e994-e1004. doi:10.1016/S2468-2667(22)00110-4

28. Ma H, Wang X, Xue Q, et al. Cardiovascular Health and Life Expectancy Among Adults in the United States. *Circulation*. 2023;147(15):1137-1146. doi:10.1161/CIRCULATIONAHA.122.062457

29. Yang Y, Chen L, Filippidis FT. Accelerometer-measured physical activity, frailty, and all-cause mortality and life expectancy among middle-aged and older adults: a UK Biobank longitudinal study. *BMC Medicine*. 2025/02/27 2025;23(1):125. doi:10.1186/s12916-025-03960-z

30. Doherty A, Jackson D, Hammerla N, et al. Large Scale Population Assessment of Physical Activity Using Wrist Worn Accelerometers: The UK Biobank Study. *PLOS ONE*. 2017;12(2):e0169649. doi:10.1371/journal.pone.0169649

31. Stamatakis E, Koemel NA, Biswas RK, et al. Minimum and optimal combined variations in sleep, physical activity, and nutrition in relation to all-cause mortality risk. *BMC Medicine*. 2025;23(1):111. doi:10.1186/s12916-024-03833-x

32. Fadnes LT, Celis-Morales C, Økland J-M, et al. Life expectancy can increase by up to 10 years following sustained shifts towards healthier diets in the United Kingdom. *Nature Food*. 2023/11/01 2023;4(11):961-965. doi:10.1038/s43016-023-00868-w

33. Health Of, Improvement, Disparities. *Annex C: data on the distribution, determinants and burden of non-communicable diseases in England*. 2021.

34. Li Y, Schoufour J, Wang DD, et al. Healthy lifestyle and life expectancy free of cancer, cardiovascular disease, and type 2 diabetes: prospective cohort study. *BMJ*. 2020;368:l6669. doi:10.1136/bmj.l6669

35. Li Y, Pan A, Wang DD, et al. Impact of Healthy Lifestyle Factors on Life Expectancies in the US Population. *Circulation*. 2018;138(4):345-355. doi:doi:10.1161/CIRCULATIONAHA.117.032047

36. Sun Q, Hu Y, Yu C, et al. Healthy lifestyle and life expectancy free of major chronic diseases at age 40 in China. *Nature Human Behaviour*. 2023;7(9):1542-1550. doi:10.1038/s41562-023-01624-7

37. Naudin S, Viallon V, Hashim D, et al. Healthy lifestyle and the risk of pancreatic cancer in the EPIC study. *European Journal of Epidemiology*. 2020;35(10):975-986. doi:10.1007/s10654-019-00559-6

38. Hautekiet P, Saenen ND, Martens DS, et al. A healthy lifestyle is positively associated with mental health and well-being and core markers in ageing. *BMC Medicine*. 2022;20(1):328. doi:10.1186/s12916-022-02524-9

39. Zhuang P, Liu X, Li Y, et al. Effect of Diet Quality and Genetic Predisposition on Hemoglobin A(1c) and Type 2 Diabetes Risk: Gene-Diet Interaction Analysis of 357,419 Individuals. *Diabetes Care*. 2021;44(11):2470-2479. doi:10.2337/dc21-1051

40. Bohlscheid-Thomas S, Hoting I, Boeing H, Wahrendorf J. Reproducibility and relative validity of food group intake in a food frequency questionnaire developed for the German part of the EPIC project. European Prospective Investigation into Cancer and Nutrition. *International journal of epidemiology*. 1997;26(suppl_1):S59.

41. Kaaks R, Slimani N, Riboli E. Pilot phase studies on the accuracy of dietary intake measurements in the EPIC project: overall evaluation of results. European Prospective Investigation into Cancer and Nutrition. *Int J Epidemiol*. 1997;26 Suppl 1:S26-36. doi:10.1093/ije/26.suppl_1.s26

42. Ocké MC, Bueno-de-Mesquita HB, Goddijn HE, et al. The Dutch EPIC food frequency questionnaire. I. Description of the questionnaire, and relative validity and reproducibility for food groups. *Int J Epidemiol*. 1997;26 Suppl 1:S37-48. doi:10.1093/ije/26.suppl_1.s37

43. Greenwood DC, Hardie LJ, Frost GS, et al. Validation of the Oxford WebQ Online 24-Hour Dietary Questionnaire Using Biomarkers. *Am J Epidemiol*. Oct 1 2019;188(10):1858-1867. doi:10.1093/aje/kwz165

44. Kim JH. Multicollinearity and misleading statistical results. *Korean J Anesthesiol*. Dec 2019;72(6):558-569. doi:10.4097/kja.19087

45. Bradbury KE, Young HJ, Guo W, Key TJ. Dietary assessment in UK Biobank: an evaluation of the performance of the touchscreen dietary questionnaire. *Journal of Nutritional Science*. 2018;7:e6. doi:10.1017/jns.2017.66

46. Arnold M, Bennett D, Bradbury K, et al. Reproducibility of dietary intakes of macronutrients, specific food groups, and dietary patterns in 211 050 adults in the UK Biobank study. *Journal of Nutritional Science*. 2019;8:e34. e34. doi:10.1017/jns.2019.31

47. Chang K, Gunter MJ, Rauber F, et al. Ultra-processed food consumption, cancer risk and cancer mortality: a large-scale prospective analysis within the UK Biobank. *eClinicalMedicine*. 2023;56doi:10.1016/j.eclinm.2023.101840

48. Monteiro CA, Cannon G, Levy RB, et al. Ultra-processed foods: what they are and how to identify them. *Public Health Nutrition*. 2019;22(5):936-941. doi:10.1017/s1368980018003762
